# Deletion of *lrrk2* causes early developmental abnormalities and age-dependent increase of monoamine catabolism in the zebrafish brain

**DOI:** 10.1371/journal.pgen.1009794

**Published:** 2021-09-13

**Authors:** Stefano Suzzi, Reiner Ahrendt, Stefan Hans, Svetlana A. Semenova, Avinash Chekuru, Paul Wirsching, Volker Kroehne, Saygın Bilican, Shady Sayed, Sylke Winkler, Sandra Spieß, Anja Machate, Jan Kaslin, Pertti Panula, Michael Brand

**Affiliations:** 1 Center for Molecular and Cellular Bioengineering (CMCB), Center for Regenerative Therapies Dresden (CRTD), Technische Universität (TU) Dresden, Dresden, Germany; 2 Department of Anatomy, Faculty of Medicine, University of Helsinki, Helsinki, Finland; 3 Max Planck Institute of Molecular Cell Biology and Genetics (MPI-CBG), Dresden, Germany; University of Pennsylvania School of Medicine, UNITED STATES

## Abstract

LRRK2 gain-of-function is considered a major cause of Parkinson’s disease (PD) in humans. However, pathogenicity of LRRK2 loss-of-function in animal models is controversial. Here we show that deletion of the entire zebrafish *lrrk2* locus elicits a pleomorphic transient brain phenotype in maternal-zygotic mutant embryos (mzLrrk2). In contrast to *lrrk2*, the paralog gene *lrrk1* is virtually not expressed in the brain of both wild-type and mzLrrk2 fish at different developmental stages. Notably, we found reduced catecholaminergic neurons, the main target of PD, in specific cell populations in the brains of mzLrrk2 larvae, but not adult fish. Strikingly, age-dependent accumulation of monoamine oxidase (MAO)-dependent catabolic signatures within mzLrrk2 brains revealed a previously undescribed interaction between LRRK2 and MAO biological activities. Our results highlight mzLrrk2 zebrafish as a tractable tool to study LRRK2 loss-of-function *in vivo*, and suggest a link between LRRK2 and MAO, potentially of relevance in the prodromic stages of PD.

## Author summary

Parkinson’s disease is the second most common degenerative disorder of the brain. Mutations of the *LRRK2* gene are the most recurrent genetic cause of pathology, and are thought to result in a more active LRRK2 protein, a large enzyme whose biological function is unclear. Therefore, LRRK2 inhibitors are regarded as promising therapeutics. However, mouse models do not reproduce human pathology unless they also lack LRRK1, and there is evidence of dominant negative effects of *LRRK2* mutations. Therefore, the characterization of reliable *LRRK2* knockout models might provide insights. In our study, we used the zebrafish as a tractable model to study both early developmental and adult phenotypes resulting from the loss of the entire endogenous *lrrk2* gene. We found that mutant embryos displayed subtle brain phenotypes, including reduction of catecholaminergic neurons, the main target of human disease, that spontaneously resolved with development, and a late-onset and progressive increase of dopamine and serotonin degradation consistent with increased MAO enzyme activity. Our results suggest that similar defects might occur in the pre-symptomatic stage of the disease in humans, and warrant further evaluation of LRRK2 inhibition in a therapeutic perspective.

## Introduction

The leucine-rich repeat kinase 2 (LRRK2) is a large multidomain protein and a bifunctional enzyme, displaying both kinase and GTPase activities [1]. *LRRK2* gene polymorphisms are the most recurrent genetic cause of familial and sporadic late-onset Parkinson’s disease (PD), one of the most common neurodegenerative disorders [2]. *In vitro* evidence has initially suggested that pathogenic variants, including the relatively common G2019S substitution, confer toxicity via gain-of-function (GOF) of the kinase domain [3,4]. Consequently, pharmacological inhibition of LRRK2 attracts great interest in a therapeutic perspective, a contention supported by the purported non-pathogenicity of loss-of-function (LOF) variants in humans [5–8]. However, the mechanisms of pathogenicity of human LRRK2 variants are still unclear. First, the current lack of reliable endogenous substrates or interaction partners makes it difficult to validate *in vitro* findings *in vivo*. More critically, mice overexpressing human wild-type or mutant LRRK2 do not generally recapitulate dopaminergic cell loss, a cardinal feature of the human disease, unless transgene levels are artificially enhanced using strong promoters [9–19]. Paradoxically, enhanced LRRK2 activity in G2019S knockin mice confers a hyperkinetic phenotype and seems protective against age-related motor impairment [20]. Finally, the hypothesis of LRRK2 LOF phenotypes resulting from dominant negative effects of established GOF mutations cannot be completely ruled out. The G2385R mutation, a risk factor for PD in the Chinese ethnicity [21,22], reduces kinase activity [23,24] and enhances LRRK2 degradation [23]. A similar dominant negative effect has been described also for the I2020T GOF variant [25,26]. Additional dominant negative effects may result from the disruption of protein-protein interactions between LRRK2 and essential partners in cell signaling pathways, as demonstrated for the pathogenic R1441C/G/H, Y1699C, and I2020T GOF variants [27]. Notably, both Lrrk LOF and overexpression of the human G2019S GOF variant have been demonstrated to impair efficient synaptic vesicle formation and endocytosis in drosophila [28].

These observations emphasize the importance of knockout studies to further elucidate LRRK2 function *in vivo*. First, they will help clarifying potential adverse effects of LRRK2 inhibition as a treatment for PD. Secondly, they might still provide insights into PD pathobiology. Interestingly, knockout of *LRRK2* gene in mice and rats yields abnormal phenotypes in peripheral organs [29–32], but no dopaminergic neurodegeneration or other neuropathological changes [31,33]. Nonetheless, LRRK2 deficiency in mice alters exploratory and motor coordination behaviors, suggesting that LRRK2 LOF must have an impact on brain function [33].

Intriguingly, increased dopamine turnover has been reported in asymptomatic human LRRK2 mutation carriers, a condition that might reflect pre-symptomatic disease [34]. Similarly, in LRRK2 models, changes in the relative abundance of dopamine and other monoamine neurotransmitters, and their catabolites, within the brain might precede microscopic alterations of the neuroanatomy–which might even never have time to develop, given the shorter lifespan of animal models relative to humans. It has been previously shown that the levels of dopamine and related catabolites are normal in the *corpus striatum* of mice lacking endogenous [31,33] or overexpressing human wild-type LRRK2 [13], but reduced in mice expressing human LRRK2 GOF variants [12,13]. However, these results may have been influenced by the choice of the experimental endpoint [31,33], the brain region [12,13], or the genetic model [13]. Moreover, the monoamine signatures reported in the LRRK2 GOF mouse models are difficult to interpret, because they are not clearly linked to problems in either dopamine synthesis [12] or turnover [12,13]. Therefore, the characterization of the monoamine fingerprint in LRRK2 models, preferably at different time points, might provide crucial insights.

The zebrafish is a valuable alternative to rodent models due to its amenability to high-throughput studies and direct observation of developmental phenotypes and disease mechanisms *in vivo*. Several attempts to investigate zebrafish *lrrk2* gene function using morpholino oligonucleotides (MOs) yielded contradicting results. In the first of such studies, loss of diencephalic catecholaminergic (CA) neurons and locomotor defects in larvae were described [35]. However, an independent group subsequently failed to reproduce these results, even by using the same reagents and MOs [36]. Subsequently, a third paper rekindled the initial claims, describing a *lrrk2* MO-induced phenotype with macroscopic developmental abnormalities [37]. Although the analysis of MO-induced phenotypes may still provide useful information, the inherent risk of off-target effects inevitably requires their validation in reliable null models [38]. In this direction, the same group who characterized the first zebrafish *lrrk2* knockdown [35] in a second study used programmable zinc-finger nucleases to generate a stable *lrrk2*-null line [39]. In their work, the authors state that the CA phenotype and locomotive activity in these mutants are inconsistent with the knockdown phenotype previously described, but only show supporting data for the adult behavior [39]. More recently, another group used the clustered regularly interspaced short palindromic repeats (CRISPR)/CRISPR-associated protein-9 nuclease (Cas9) genome-editing tool and generated three zebrafish *lrrk2* alleles that are suggested to lack either some or all of the functional domains [40]. Although all three mutants have normal morphology and locomotion, phenotypic characterization is limited to the embryonic stage [40]. Notably, none of the mutants show evidence of nonsense-mediated decay of *lrrk2* mRNA [40], which leaves it open that the mutant transcripts might still yield functional products.

Given the controversy sparked by the three extant *lrrk2* knockdown studies, and the ambiguity as to whether age-dependent deficits of relevance for PD occur in adult *lrrk2*-null mutants, a more comprehensive and thorough characterization of the zebrafish *lrrk2*-null phenotype is warranted. In particular, in addition to neuroanatomic changes, it is critical to evaluate possible functional consequences. Specifically, since brain monoamine neurochemistry is conserved between zebrafish and mammals [41], the evaluation of the monoamine fingerprint in zebrafish *lrrk2* mutants, possibly at different time points, may be revealing of subtler, age-dependent functional alterations of relevance for a slowly progressing disease such as PD.

Here, we used CRISPR/Cas9 to delete the ~60-kilobase pairs-long zebrafish *lrrk2* locus containing the entire open reading frame (ORF), thus generating a *bona fide* null allele. We characterized the phenotype of the brain as the primary organ affected by PD. We found that maternal-zygotic *lrrk2* (mzLrrk2) mutant larvae display a pleomorphic but transient phenotype indicative of developmental delay. We verified that, throughout development, the paralog gene *lrrk1* maintains almost non-overlapping expression domains compared to *lrrk2*, being virtually not expressed in the brain, and is not upregulated in mzLrrk2 mutants. Interestingly, distinct CA cell populations in the brain are specifically reduced in the larvae, but not in the adults. Strikingly, however, we found a progressive increase of monoamine oxidase (MAO) enzyme-dependent monoamine catabolism in adulthood. Our results suggest a link between zebrafish Lrrk2 and MAO-dependent monoamine catabolism, and that its perturbation may lead to progressive neurochemical defects akin to human PD patients.

## Results

### Deletion of the entire *lrrk2* locus using CRISPR/Cas9

**Fig 1:**
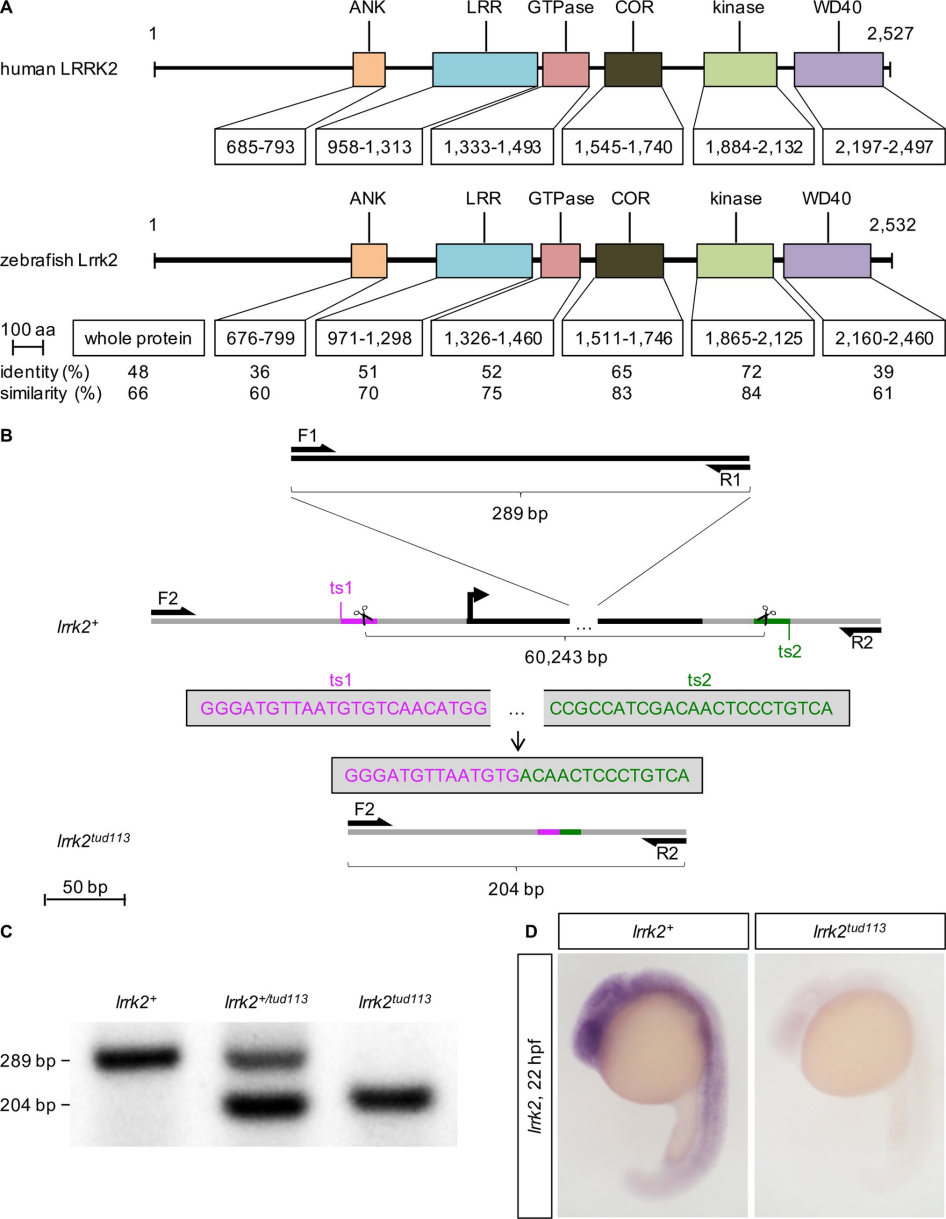
Homology between the human LRRK2 and the zebrafish Lrrk2 proteins and generation of the zebrafish *lrrk2*^*tud113*^ allele. (A) Alignment of the whole sequence and the individual domains of human LRRK2 (NP_940980) and zebrafish Lrrk2 (NP_001188385) proteins revealed a high degree of conservation of the catalytic core. The percentages of identity (same residues at the same positions in the alignment) and similarity (identical residues plus conservative substitutions) are indicated. Abbreviations: aa, amino acids; ANK, ankyrin domain; COR, C-terminal of Ras of complex proteins; LRR, leucine-rich repeat domain. (B) Scheme reproducing the *lrrk2* gene targeting and screening strategy. The *lrrk2* open reading frame (ORF) is highlighted in black; F1, R1: *lrrk2 ORF*-specific primers; F2, R2: *lrrk2 ORF*-flanking primers; ts1 (magenta), ts2 (green): gRNA target sites (ts); bp, base pairs. (C) gap-PCR analysis of genomic DNA from wild-type (*lrrk2*^*+*^), heterozygous (*lrrk2*^*+/tud113*^), and homozygous mutant (*lrrk2*^*tud113*^) individuals. F1 and R1 amplify a 289-base pairs-long product, F2 and R2 a 204-base pairs-long product. (D) *lrrk2* ISH confirming the complete absence of *lrrk2* expression in 22-hpf *lrrk2*^*tud113*^ embryos.

Syntenic analysis using the assemblies of the human and zebrafish genomes (GRCh38p.7 and GRCz10, respectively) revealed the conservation of *SLC2A13* as the downstream neighbor of *LRRK2* in both species. Moreover, duplication of the zebrafish *lrrk2* locus is not reported. The human LRRK2 and zebrafish Lrrk2 proteins share the same domains, with the kinase domain displaying the highest degree of conservation (Fig 1A); three of the four sites of pathogenic substitutions in humans are fully conserved (Table 1). Altogether, these data point out that human *LRRK2* and zebrafish *lrrk2* are true orthologs, and not divergent genes.

**Table 1:** Sites of pathogenic amino acid substitutions in human LRRK2 and corresponding residues in zebrafish Lrrk2.

substitution in humans	corresponding site in zebrafish	protein domain
p.Arg1441Cys		
p.Arg1441Gly		
p.Arg1441His		
p.Tyr1699Cys	Tyr1685	COR
p.Gly2019Ser	Gly2009	kinase
p.Ile2020Thr	Ile2010	kinase

Data retrieved from the Parkinson Disease Mutation Database (PDmutDB, http://www.molgen.vib-ua.be/PDmutDB) [6,42].

During zebrafish development, *lrrk2* expression is ubiquitous until 24 hours post-fertilization (hpf), then gradually becomes restricted to the head (S1 Fig). In the adult brain, *lrrk2* expression is present throughout the whole organ, including the regions containing CA nuclei, such as the olfactory bulb, the ventral telencephalon, the pretectal area, and the ventral diencephalon (S2 Fig). The time course of *lrrk2* expression in the zebrafish brain thus closely mirrors the one in rodents, where *Lrrk2* is broadly expressed in the embryonic, postnatal, and adult brain, though at low levels compared to other peripheral tissues [43–45].

Previous studies on MO-induced *lrrk2* knockdown in zebrafish have yielded contradicting findings [35–37]. We independently designed and tested MOs but, although *lrrk2* knockdown was successful (S3 Fig), we could detect no phenotype, similarly to one of the published studies [36]. We next sought to resolve the controversy by generating a stable zebrafish *lrrk2*-null line. With this motivation, we combined *N*-ethyl-*N*-nitrosourea-mutagenesis with reverse genetics screening [46] and identified a *lrrk2* allele that carries a point mutation (c.3972+2T>C according to the Human Genome Variation Society guidelines [47]; henceforth referred to as “*tud112*”) causing a premature stop within the LRR domain-coding region (p.(Ile1252AlafsTer9); S4A–S4C Fig). Preliminary characterization of the maternal-zygotic phenotype (mzLrrk2^tud112^) revealed interesting developmental defects, including reduced expression of *th1*, a marker of CA neurons, during development (S4D Fig). However, the existence of background mutations due to the chemical mutagenesis could not be ruled out.

In an effort to generate a *bona fide* zebrafish *lrrk2*-null line, we then sought to remove the *lrrk2* locus containing the entire ORF. This approach overcomes potential side effects of frameshift mutations, including cellular stress due to aberrant transcripts and truncated protein products with residual or new function. To achieve full deletion of the *lrrk2* locus, two CRISPR/Cas9 target sites flanking one 75 base pairs upstream the ATG codon, the other 33 base pairs downstream the TAA stop codon were chosen (Fig 1B). To identify deletion alleles, a gap-PCR strategy was devised, with primers amplifying a 289-base-pairs-long amplicon inside the target region duplexed with flanking primers, unable to direct amplification unless the deletion brings them into sufficient reciprocal proximity (Fig 1B and 1C). The selected founder produced offspring where the flanking primers amplified a 204-base-pairs-long product, revealing a 60,243-base-pairs-long targeted deletion (c.−61_*42del; henceforth this allele is referred to as “*tud113*”) as confirmed by sequencing. The complete absence of *lrrk2* expression in homozygous *tud113* mutants was verified via *in situ* hybridization (ISH; Fig 1D).

F1 heterozygous fish were incrossed to obtain F2 homozygous *tud113* mutants and wild types (wt). To exclude any effect from the existing maternal wt *lrrk2* transcripts (S1A Fig), F2 fish were further incrossed to obtain homozygous mutants that developed from homozygous mothers (maternal-zygotic homozygous *tud113* mutants, henceforth referred to as “mzLrrk2”). In striking contrast with published MO-induced phenotypes [35,37], mzLrrk2 individuals developed normally, were viable, and reached sexual maturity at the same age as controls, with both females and males being fertile.

### Pleomorphic brain phenotype in mzLrrk2 larvae

**Fig 2:**
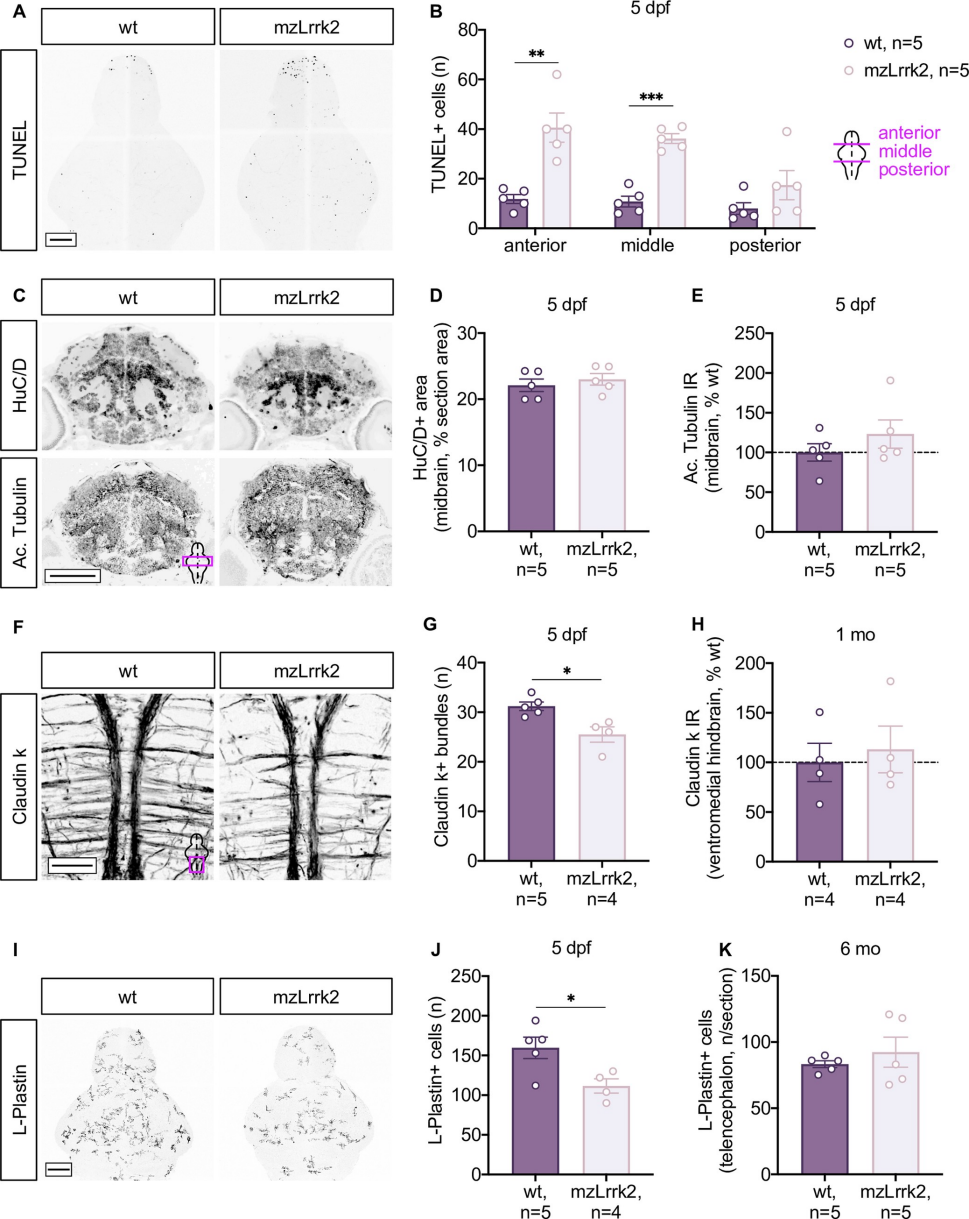
Early but transient neurodevelopmental phenotypes in mzLrrk2 larvae. (A and B) Increased cell death in the brains of mzLrrk2 larvae (5 dpf) as revealed by TUNEL assay. Quantification was carried out over the whole brain, subdivided into anterior (telencephalon), middle (diencephalon, mesencephalon), and posterior (rhombencephalon) portions. (A) Scale bar: 100 μm. (B) Plot represents means ± s.e.m. Statistical analyses: two-tailed Student’s *t*-test. (C-E) No evidence of midbrain neurodegeneration in mzLrrk2 larvae (5 dpf). HuC/D IHC (C-top and D) and acetylated (Ac.) Tubulin IHC (C-bottom and E) were used to label postmitotic neurons and axonal network. (C) Scale bar: 100 μm. (E) IR, immunoreactivity (measured as integrated density). (D and E) Plots represent means ± s.e.m. Statistical analyses: two-tailed Student’s *t*-test. (F-H) Claudin k+ myelinated commissural fibers in the ventral hindbrain were reduced in mzLrrk2 larvae (5 dpf; F and G), but not in youngsters (1 mo; H; representative images in S6 Fig). (F) Scale bar: 100 μm. (H) IR, immunoreactivity (measured as integrated density). (G and H) Plots represent means ± s.e.m. Statistical analyses: two-tailed Student’s *t*-test. (I-K) L-Plastin+ microglia/leukocytes were reduced in the brains of mzLrrk2 larvae (5 dpf; I and J), but not in the brains of adult fish (6 mo; K, quantification in the telencephalon as representative brain region; representative images in S8 Fig). (I) Scale bar: 100 μm. (J) Quantification of microglia/leukocytes in the larval brain was performed after image segmentation as depicted in S7A–S7D Fig. (J and K) Plots represent means ± s.e.m. Statistical analyses: two-tailed Student’s *t*-test.

Since the human LRRK2 phenotypes are age-dependent, we characterized the brain phenotype of mzLrrk2 fish at both larval (5 days post-fertilization, dpf) and adult stages. Using the TUNEL assay, we measured a threefold increase of the cell death rate in mzLrrk2 larval brains (anterior portion: *P* = 0.0061; middle portion: *P*<0.0001; Fig 2A and 2B). Previous *lrrk2* knockdown studies have reported neuronal loss in MO-injected embryos [35,37]. In particular, the midbrain was described as specifically affected [35]. Therefore, we used HuC/D and acetylated Tubulin immunohistochemistry (IHC) to visualize postmitotic neurons and axonal network and specifically focused on the midbrain. In contrast to the published MO phenotype [35], we did not find differences between mzLrrk2 larvae and wt controls (Fig 2C–2E).

Several lines of evidence implicate LRRK2 in cell proliferation and differentiation [48]. Impaired neurogenesis and neurite outgrowth were reported in transgenic mice expressing the human G2019S variant [49]. Knockout studies have led to ambiguous findings: in mice, the number of DCX+ neuroblasts in the dentate gyrus was reported to increase in one study [50], but was unaffected according to another [33]. Using phospho-histone H3 (pH3) as a marker for mitotic cells, we found that the number of pH3+ cells in the larval brain was comparable between mzLrrk2 and wt controls (mzLrrk2, n = 5: 277.0±10.7; wt, n = 6: 281.3±10.9; *P* = 0.7853). To address more specifically neurogenesis at a later time point, we performed an EdU-based pulse-chase assay on juvenile fish of 1 month of age (mo) and found normal production of new neurons (S5 Fig). Altogether, these data indicate that embryonic neurogenesis is overall not affected by loss of *lrrk2*.

To further investigate neural development, we next inspected the non-neuronal compartment. Using Claudin k IHC, we quantified myelinated tracts in the ventromedial hindbrain and found a marked reduction (Fig 2F and 2G). However, when we analyzed youngsters (1 mo), Claudin k immunoreactivity was not different between mzLrrk2 fish and wt controls (Figs S6 and 2H), indicating that the phenotype was transient. Microglia play important roles in numerous developmental processes, including myelination [51]. Upon activation, microglia express high levels of *LRRK2* [52]. Interestingly, weakened antibacterial response was reported in a *lrrk2*-null zebrafish line [39]. Reasoning that impaired neuroimmune functions might affect developmental dynamics, we examined the number of microglia/leukocytes in mzLrrk2 larval brains (S7A–S7E Fig) and found an overall reduction of about one third (Fig 2I and 2J; *P* = 0.0273). Additionally, mzLrrk2 microglia/leukocytes displayed slight, but significant morphological differences, such as larger volume (*P* = 0.0318, S7F Fig) and surface (*P* = 0.0180, S7G Fig) and higher ramification (*P* = 0.0142, S7H Fig), which might be indicative of altered levels of activation. In contrast, the numbers of microglia/leukocytes in the adult brains (6 mo) were comparable between mzLrrk2 fish and age-matched wt controls (Figs S8 and 2K), suggesting phenotype attenuation during development.

In summary, our data show that loss of *lrrk2* does not cause overt alterations of brain structure, but rather subtle transient phenotypes compatible with general neurodevelopmental delay. However, the larval microglia/leukocyte phenotype, which suggests altered immune resilience, while apparently resolving in adulthood, might still manifest under immune challenge [39].

### Loss of *lrrk2* does not affect spontaneous swimming ability, anxiety, and olfaction

PD patients are characterized by a motor syndrome comprising resting tremor, rigidity, postural instability, and slowness of movement. The initial *lrrk2* knockdown study in zebrafish reported reduced swum distance in MO-injected larvae [35]. The same authors later characterized a *lrrk2*-null line and described hyperactivity [39]. In both studies, however, swimming activity was assessed for only 30 s, and no information about habituation time or environmental context was provided. The second *lrrk2* knockdown study described normal swimming ability [36].

To clarify whether loss of *lrrk2* affects zebrafish locomotion, spontaneous swimming activity of 5-dpf larvae and 6-mo adult fish was automatically recorded for 10 min and analyzed. For each recording, a total of six motor parameters for the larvae, and four for the adults were considered. The statistics for the individual parameters are reported in Table 2. As can be seen, the locomotive activity was comparable between mzLrrk2 individuals and wt controls. In the larvae, although the inactive phase duration was significantly longer in the mutants, the median difference was only 16.2 sec over a 10-min recording, and thus probably not biologically relevant. Altogether, the data indicate that mzLrrk2 fish have intact swimming ability throughout development.

**Table 2:** Analysis of spontaneous swimming activity.

time point	motor parameter	mzLrrk2	wt	*P*
5 dpf	inactive phase duration (s)	374.5±7.3	354.5±7.2	0.0162
	inactive phase distance (cm)	136.6±4.3	141.8±4.5	
	normal phase duration (s)	198.0±6.8	208.1±6.0	
	normal phase distance (cm)	1141.0±131.2	1104.0±99.3	
	bursting phase duration (s)	8.9±2.1	9.9±2.3	
	bursting phase distance (cm)	362.5±167.3	308.5±118.8	
6 mo	normal phase duration (s)	398.6±9.3	406±12.8	
	normal phase distance (cm)	13009.3±292.9	12216.3±303.7	
	bursting phase duration (s)	197.9±9.3	191.6±12.9	
	bursting phase distance (cm)	12432.3±632.6	11682.0±850.0	

Data express means ± s.e.m. Sample size: 5 dpf mzLrrk2, n = 58; 5 dpf wt, n = 53; 6 mo mzLrrk2, n = 91; 6 mo wt, n = 66. Statistical analyses: Mann-Whitney’s *U*-test. *P*<0.0500 only reported.

Several non-motor symptoms often precede motor disease and aggravate disability in later stages of PD pathology. Anxiety and olfactory dysfunction are amongst the most prevalent in PD patients both after and prior to diagnosis [53]. Therefore, we sought to analyze anxiety levels and response to olfactory stimuli in fish. To test anxiety levels, two parameters were quantified: 1) wall-hugging behavior, or thigmotaxis, for both larvae (5 dpf) and adults (6 mo; S9A–S9C Fig), and 2) dark-to-light preference, or scototaxis, for adults only (S9D Fig). To evaluate the overall olfactory function in adult fish, the response to an amino acid mixture odorant stimulus was measured (S9E Fig). None of the assays revealed significant differences between mzLrrk2 fish and controls. Altogether, our data indicate that loss of *lrrk2* in zebrafish does not result in macroscopic deviations of both motor and non-motor behaviors of relevance for PD.

### The paralog *lrrk1* is not upregulated due to genetic compensation in mzLrrk2 fish

The autosomal dominant nature of human *LRRK2* mutations and the relatively subtle *lrrk2*-null phenotypes so far described raise the question whether, in mzLrrk2 embryos, genes functionally related to *lrrk2* undergo compensatory upregulation during development [38]. In mice, the paralog gene *Lrrk1* has been speculated to compensate *Lrrk2* knockout [31,54]. However, this contention is controversial. On the one hand, *Lrrk2*/*Lrrk1* double knockout mice develop age-dependent PD-like neuropathology, including selective CA neurodegeneration [55]. On the other hand, there is substantial evidence that *Lrrk1* and *Lrrk2* are divergent genes with different functions. First, *Lrrk1* and *Lrrk2* genes have non-overlapping expression domains in the mouse embryo and adult mouse brain [43]. Second, the *Lrrk1* and *Lrrk2* knockout phenotypes in mice are distinct, with loss of *Lrrk1* seemingly having no impact on brain function, but causing a peculiar bone phenotype instead [56]. Accordingly, the *Lrrk2*/*Lrrk1* double knockout phenotype may be the result of reduced organismic resilience due to compound phenotypes, as suggested by the inherent young mortality [55], in contrast to the slowly progressive nature of PD pathology in humans. Finally, LRRK1 and LRRK2 proteins have different protein-protein interactions [57].

Given the conflicting evidence above, we sought to extend the genetic analysis of the mzLrrk2 zebrafish model by considering the possibility of compensation by Lrrk1. To approach this, we first compared zebrafish Lrrk1 and Lrrk2 protein sequences and found little homology (S10A Fig). Of note, the homology between the two fish paralog proteins was significantly lower than between the zebrafish Lrrk2 and the human LRRK2 protein orthologs (Fig 1A). Second, we characterized *lrrk1* gene expression in developing wt embryos and found that *lrrk1* was expressed at much lower levels than *lrrk2* and in specific tissue-restricted domains (S10B–S10J and S1 Figs). Notably, with the exception of the sensory organs (S10F–S10J and S1K Figs), *lrrk1* and *lrrk2* were expressed in distinct domains. In particular, in contrast to *lrrk2*, which was strongly expressed in the developing nervous system (S1 Fig), *lrrk1* was only transiently expressed in few neural structures in the embryo, specifically in the midbrain-hindbrain boundary at 20 ss (S10D Fig) and in the telencephalon and ventral hindbrain at 24 hpf (S10E Fig), but not at 32–48 hpf (S10F–S10J Fig). Taken together, these data suggest that zebrafish Lrrk1 and Lrrk2 have largely non-overlapping biological functions.

Next, we addressed the expression of *lrrk1* upon loss of *lrrk2*. First, we measured the levels of *lrrk1* mRNA during oogenesis in transheterozygous *tud113/tud112* females. Using RT-qPCR, we found that *lrrk1* transcripts were downregulated in *lrrk2*^–^ oocytes compared to *lrrk2*^+^ controls (*P* = 0.0019; S11A Fig). However, embryos derived from an incross of transheterozygotes showed normal *lrrk1* transcript levels upon ISH (S11B Fig). Similarly, in mzLrrk2 larvae (5 dpf), the expression level of *lrrk1* was overall unaltered in the whole body as determined by RT-qPCR (S11C Fig). Furthermore, inspection of the brain via ISH at different developmental stages–larva (5 dpf), youngster (1 mo), and adult (6.5 mo)–revealed that expression of *lrrk1*, in stark contrast to that of *lrrk2* (S2 Fig), remained below detection in both wt and mzLrrk2 fish, despite the long chromogenic signal development time applied (72 h; S11D and S11E Fig). In sum, at developmental stages and in brain regions where *lrrk2* is normally expressed, *lrrk1* is virtually absent and its expression does not change upon loss of *lrrk2*.

### Specific CA defects in the larval brain are compensated in adulthood

**Fig 3:**
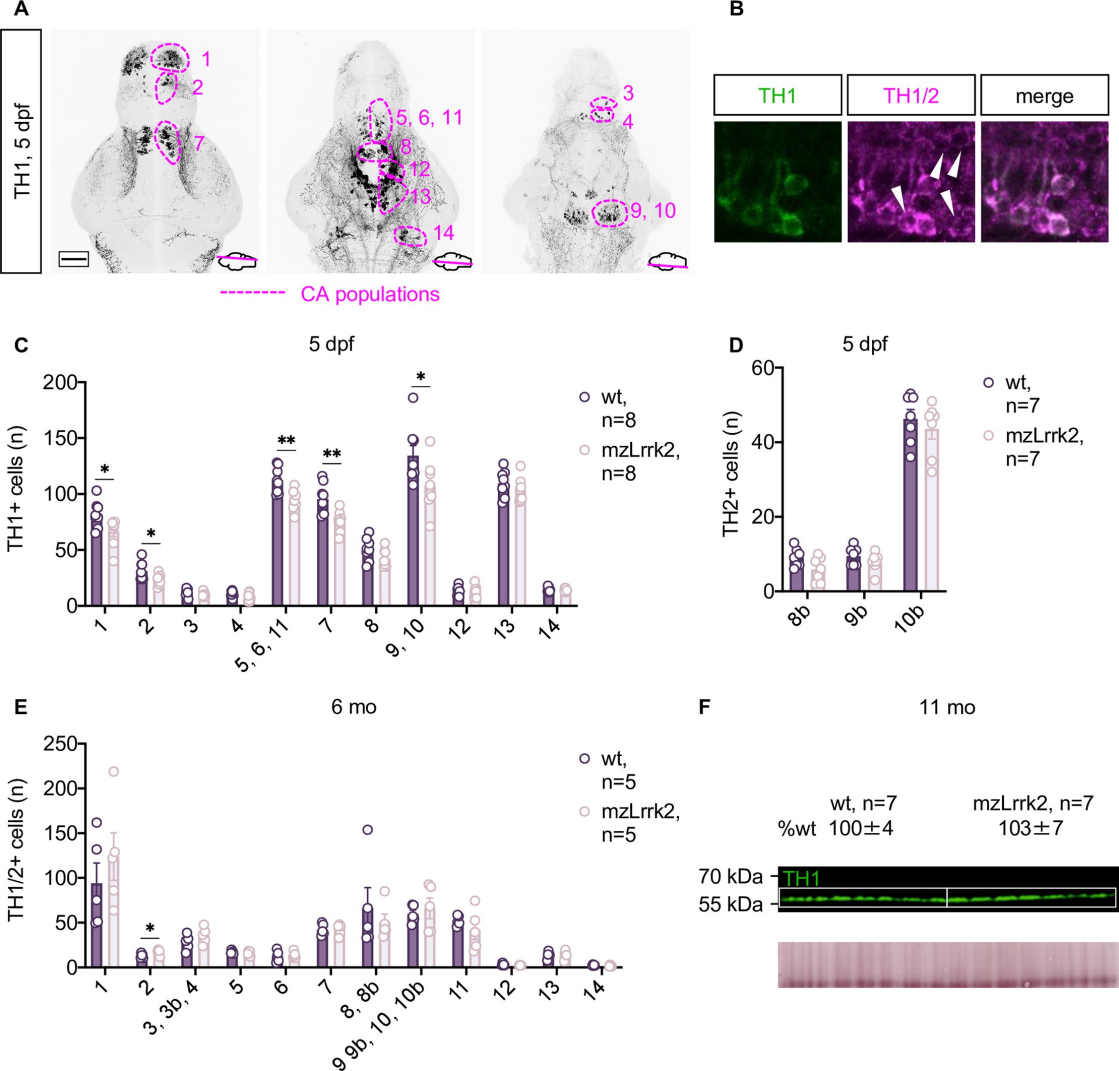
Population-specific defects in the CA system of mzLrrk2 larvae. (A) The zebrafish CA cell populations in the rostro-caudal axis from the olfactory bulb to the *locus cæruleus* as revealed by tyrosine hydroxylase 1 (TH1) IHC at 5 dpf. Pop. 1: olfactory bulb; pop. 2: telencephalic complex; pop. 3: preoptic area, *pars anterior*; pop. 4: preoptic area, *pars posterior*; pop. 5, 6, 11: diencephalic complex; pop. 7: pretectal area; pop. 8: paraventricular organ, *pars anterior*; pop. 9: paraventricular organ, *pars intermedia*; pop. 10: paraventricular organ, *pars posterior*; pop. 12: posterior tuberal nucleus/posterior tuberculum; pop. 13: hypothalamic complex; pop. 14: *locus cæruleus*. (B) Combination of the anti-TH1 antibody with the pan-TH antibody allows the identification of TH2+ cells (white arrowheads) by exclusion. TH2+ cells are found within the TH1 pop. 8, 9, and 10 in the paraventricular organ, thereby constituting the TH2 pop. 8b, 9b, 10b. (C and D) Quantification of TH1+ (C) and TH2+ cells (D) revealed defects in distinct CA cell populations in the brain of mzLrrk2 larvae (5 dpf). Plot represents means ± s.e.m. Statistical analyses: (C: pop. 2–14 and D) two-tailed Student’s *t*-test; (C: pop. 1) two-tailed Mann-Whitney’s *U*-test. (E) Quantification of TH1/2+ cells in the adult brain (6 mo) revealed absence of CA cell defects in mzLrrk2 fish. Plot represents means ± s.e.m. Statistical analyses: (E: pop.1, E: pop. 3–11, E: pop. 13 and E: pop. 14) two-tailed Student’s *t*-test; (E: pop. 2 and E: pop. 12) two-tailed Mann-Whitney’s *U*-test. (F) Quantification of TH1 protein levels in 11-mo brains shows intact CA system in adult fish; total protein stain as loading control is shown. Protein levels are reported as means ± s.e.m. relative to wt levels (%). Statistical analysis: two-tailed Student’s *t*-test.

Because CA neurons are the most clinically relevant target of PD, special attention was paid to the analysis of the CA system in mzLrrk2 fish. To this end, CA cell populations along the rostro-caudal axis from the olfactory bulb to the *locus cæruleus* were visualized via tyrosine hydroxylase (TH) IHC on whole-mounted brains [41] (Fig 3A). Zebrafish possess two paralogous *th* genes: *th1* and *th2* [58]. Because the commercially available anti-TH antibodies only recognize TH1, but not TH2 protein [59], we analyzed the zebrafish CA system by double staining with an anti-TH1 antibody and a recently characterized pan-TH antibody to also identify TH2+ cells by exclusion (Fig 3B).

Similar to our findings in mzLrrk2^tud112^ embryos (S4D Fig), we observed that no TH+ cell population was missing or overtly altered in mzLrrk2 larval brains. However, discrete cell populations displayed lower numbers of TH+ cells: olfactory bulb (pop. 1, *P* = 0.0313); telencephalic complex (pop. 2, *P* = 0.0322); diencephalic complex (pop. 5, 6, 11, *P* = 0.0042); pretectal area (pop. 7, *P* = 0.0042); and paraventricular organ, *partes intermedia* and *posterior* (pop. 9, 10 *P* = 0.0407; Fig 3C and 3D). Of interest, the diencephalic complex is considered the anatomical correlate of the mammalian *substantia nigra* [60], the major target of PD-associated neurodegeneration in humans. The population-specificity of the CA defects suggests them to be a true defect, rather than a delay. The net effect was a 20% reduction of the overall number of TH+ cells (mzLrrk2, n = 8: 557.3±19.9; wt, n = 8: 665.4±18.4; *P* = 0.0014). Because mzLrrk2 brains showed a higher cell death rate (Fig 2A and 2B), TUNEL assay was combined with TH IHC to investigate whether CA neurons were particularly affected. However, virtually no colocalization was found.

As zebrafish constantly produce neurons, we addressed whether the initial CA defects were due to impaired CA neurogenesis. To this aim, embryos (3 dpf) were soaked in BrdU to label proliferating cells and chased for TH+ neurons at 5 dpf (S12A–S12C Fig). In line with the above, the overall number of TH+ cells was lower in mzLrrk2 brains (*P* = 0.0114; S12D Fig). The proportion of TH+/BrdU+ cells was only 1–2%, reflecting a low neurogenic rate at the developmental window considered for chasing (S12E Fig). Although the number of TH+/BrdU+ neurons trended lower in mzLrrk2 brains, the difference was not statistically significant (mzLrrk2, n = 10: 4.4±2.3; wt, n = 15: 8.3±2.6; *P* = 0.2120).

In the adult brain, the CA system appeared structurally intact at 6 mo, with a modest increase in cell number in the telencephalic complex (pop. 2, *P* = 0.0472; Fig 3E). Furthermore, TH1 protein levels at 11 mo were normal (Fig 3F). In summary, despite initial defects, the CA system develops normally and is structurally stable in adulthood as checked at 6 and 11 mo. Nonetheless, it shall be noted that the embryonic CA defect may span a long time window, as we did not regularly followed CA neurons during the larval to juvenile stages.

### Progressive increase of MAO-dependent monoamine catabolism

**Fig 4:**
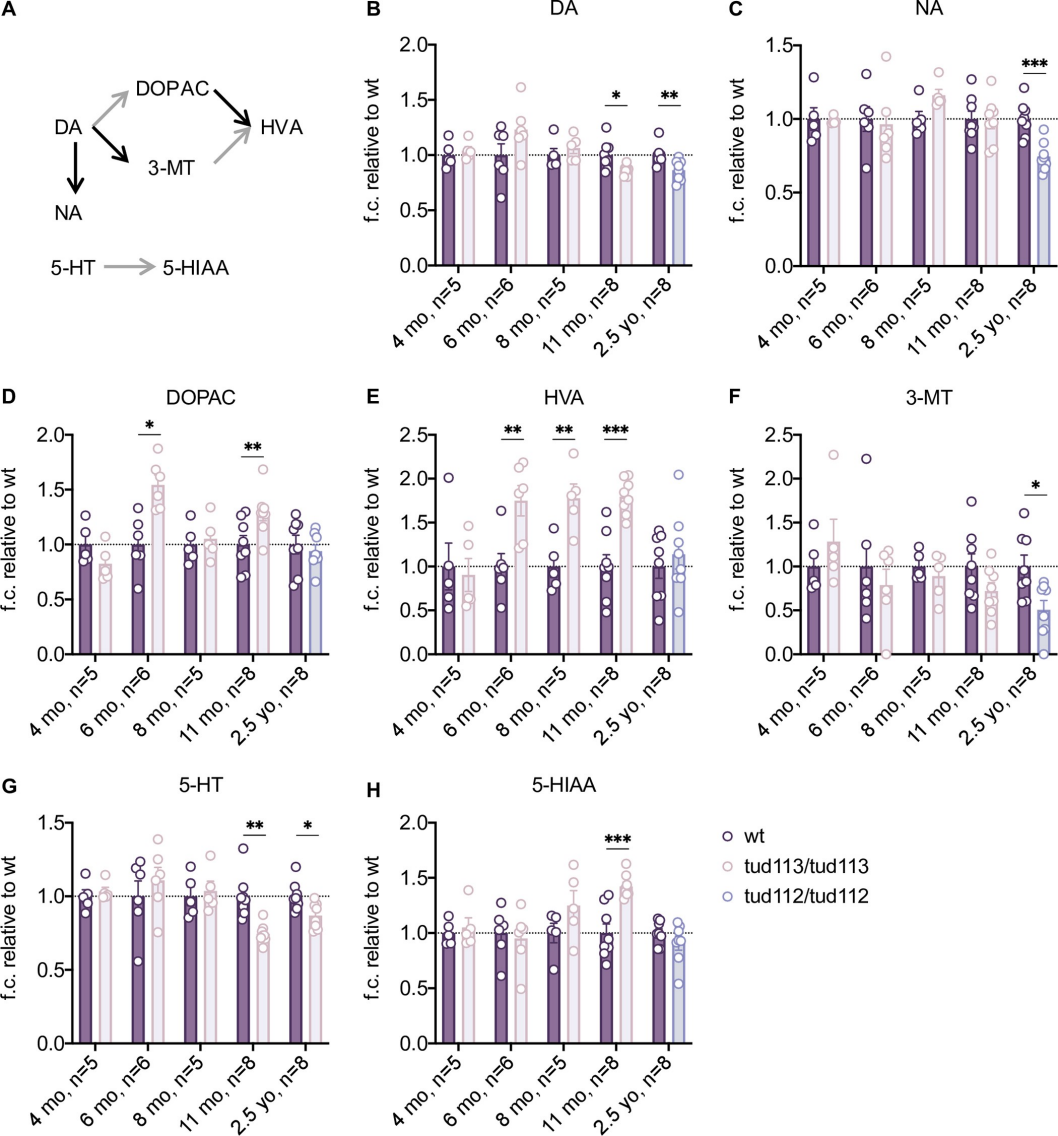
Age-dependent perturbation of monoamine catabolism in the mzLrrk2 brain. (A) Simplified scheme of the catabolism of dopamine and serotonin in the zebrafish brain. Each arrow represents a distinct enzymatically-catalyzed step. Grey arrows indicate the reactions catalyzed by the combined action of monoamine oxidase (MAO)/aldehyde dehydrogenase. Abbreviations: 3-MT, 3-methoxytyramine; 5-HIAA, 5-hydroxyindoleacetic acid; 5-HT, serotonin; DA, dopamine; DOPA, 3,4-dihydroxyphenylacetic acid; HVA, homovanillic acid; NA, noradrenalin. (B-H) Levels of biogenic amines and their catabolites in the brain of adult maternal-zygotic *tud113* fish at different ages: 4, 6, 8, and 11 months, and age-matched controls; data from 2.5-year-old (yo) maternal-zygotic *tud112* fish, and age-matched controls, are also included. Plots represent means ± s.e.m. Statistical analyses: (B: 4–6 mo, B: 11 mo, C: 6 mo, C: 11 mo, C: 2.5 yo, D-G: 6–11 mo, G: 2.5 yo, H) two-tailed Student’s *t*-test; (B: 8 mo, B: 2.5 yo, C: 4 mo, C: 8 mo, G: 4 mo) two-tailed Mann-Whitney’s *U*-test.

Despite the apparent structural integrity, the CA system of mzLrrk2 fish may be impaired on a subtler, functional level. Therefore, we measured the levels of biogenic monoamines and their catabolites via electrochemical detection coupled with high performance liquid chromatography. Analyses conducted on whole larvae at 5 dpf did not reveal perturbations of dopamine catabolism (S13 Fig). Conversely, the amine catabolism was progressively perturbed in the mzLrrk2 adult brain (Fig 4). At 4 mo, there was no difference between mzLrrk2 brains and wt controls. At 6 mo, mzLrrk2 brains contained normal dopamine, but higher concentrations of the dopamine catabolite 3,4-dihydroxyphenylacetic acid (DOPAC; *P* = 0.0029) and the dopamine/serotonin catabolite homovanillic acid (HVA; *P* = 0.0080). At 8 mo, HVA levels were still significantly higher (*P* = 0.0054). At 11 mo, dopamine and serotonin levels were significantly reduced (*P* = 0.0215 and *P* = 0.0013, respectively); correspondingly, DOPAC, HVA, and the serotonin catabolite 5-hydroxyindoleacetic (5-HIAA) acid were increased (*P* = 0.0187, *P* = 0.0001, and *P* = 0.0004, respectively). Thus, the increase of monoamine catabolism in mzLrrk2 brains resulted in a net reduction of both dopamine and serotonin after about five months from its onset, between 4 and 6 mo. The progressive nature of these neurochemical signatures suggests more severe defects at later time points. Accordingly, we found a general reduction of monoamines and metabolites in mzLrrk2^tud112^ fish at the age of 2.5 years (yo; dopamine: *P* = 0.0087; noradrenaline: *P* = 0.0004; 3-methoxytyramine: *P* = 0.0230; serotonin: *P* = 0.0137).

Interestingly, mzLrrk2 brains displayed increased levels of DOPAC, HVA, and HIAA, all products of the MAO enzyme, but normal levels of the dopamine catabolite 3-methoxytyramine, product of the catechol-O-methyltransferase (COMT) enzyme. Because MAO and COMT are the two enzymes responsible for catecholamine catabolism, the observed monoamine signatures could be ascribed to increased MAO activity. One possibility is that MAO is overexpressed in absence of Lrrk2. Unfortunately, an anti-MAO antibody working in zebrafish is currently not available, which makes direct visualization and quantification of MAO protein impossible. Therefore, we tried to estimate MAO abundance indirectly, on both transcriptional and enzymatic activity level, in the brains of fish at the age of 11 mo, i.e. at the time point when both dopamine and serotonin were found reduced in mzLrrk2 individuals (Fig 4B and 4G). Quantification of *mao* transcripts in whole brains by RT-qPCR did not reveal differences between mzLrrk2 fish and wt controls (S14A Fig). However, visualization of *mao* gene expression by ISH revealed potential local upregulation, particularly in the ventral diencephalon (S14B Fig). Potentially increased *mao* signal was also found in mzLrrk2^tud112^ fish at the age of 10.5 mo, albeit in different areas, i.e. in the ventral (S14C Fig) and dorsal telencephalon (S14C Fig, insets). Similarly to *mao* gene expression, when we measured MAO enzymatic activity in whole brains, we did not find a significant difference between mzLrrk2 fish and wt controls (S14D Fig). However, when we histochemically visualized MAO enzymatic activity in brain tissue, although less clearly than after *mao* ISH due to higher noise, we found slight differences that we interpreted as potential local upregulation in the ventral diencephalon in mzLrrk2 fish relative to wt controls (S14E Fig). Altogether, these data suggest that loss of *lrrk2* potentially leads to upregulation or redistribution of *mao* transcripts/MAO enzyme in distinct brain regions, which would be masked in bulk analyses on the whole organ. Unfortunately, because neither *mao* ISH nor MAO histochemistry are stoichiometric methods, they do not allow for quantitative assessment. Nonetheless, the qualitative histology data together with the progressive monoamine phenotype in mzLrrk2 fish suggest a previously undescribed functional linkage between Lrrk2 and MAO biological activities.

## Discussion

The homology between human LRRK2 and zebrafish Lrrk2 proteins, the temporal and regional dynamics, and entity of *lrrk2* gene expression in the zebrafish brain from early embryo to adult make the zebrafish a suitable vertebrate model to study LRRK2 biology. Here we present the *lrrk2*^*tud113*^ allele consisting in the complete deletion of the *lrrk2* locus and describe the associated brain phenotypes in maternal-zygotic mutants (mzLrrk2). The large size of the deletion, more than 60 kilobase pairs, poses a caveat: whether or not the targeted locus harbors genetic activities besides *lrrk2*, as in humans [61]. We controlled that no intragenic ORF is annotated in the latest zebrafish genome assembly (GRCz11). Of course, such possibility cannot be excluded completely. However, key phenotypic findings in maternal-zygotic *tud113* mutants, namely the CA defect early in development and the upregulation of *mao* in the adult brain, are recapitulated in maternal-zygotic *tud112* mutants, which bear a single nucleotide substitution that leaves the entire intronic sequence intact. Therefore, the mzLrrk2 phenotypes are unlikely to result from disruption of intron sequences that are unrelated to the *lrrk2* gene itself.

In contrast to published MO-induced *lrrk2* phenotypes [35,37], we found that mzLrrk2 mutants have weaker and transient developmental phenotypes. One explanation could be the activation of compensatory mechanisms triggered by the deleterious *tud113* allele, which may not have time to occur after *lrrk2* gene knockdown [38]. However, we independently performed MO-mediated knockdown of *lrrk2*, which resulted in no obvious abnormality. Our findings match those from another study [36], and altogether suggest that the MO-induced phenotypes previously reported are the result of off-target effects. Alternatively, functionally-related genes may compensate for loss of *lrrk2* later in development. We addressed this possibility for the most obvious candidate, i.e. the paralog gene *lrrk1*. We verified that Lrrk1 and Lrrk2 paralog proteins have moderate-to-low degree of homology, particularly when comparing the functional domains only, and almost non-overlapping expression domains during development. More critically, *lrrk1* is virtually not expressed in the brain throughout development, and so remains also in mzLrrk2 fish. Altogether, and in line with evidence from studies in mice, our data do not support the hypothesis that *lrrk1* can functionally replace *lrrk2* in zebrafish.

Notably, in contrast to previous *Lrrk2* knockout studies in mice [33], we show that loss of *lrrk2* in zebrafish does affect the CA system on a functional level. Specifically, we found neurochemical fingerprints compatible with enhanced MAO-dependent monoamine degradation, encompassing reduced dopamine levels in 11-mo mzLrrk2 and 2.5-yo mzLrrk2^tud112^ fish. Consistently, although we could not perform quantitative measurements due to technical limitations, we found qualitative evidence of potential local upregulation of *mao* gene expression and MAO enzyme activity in the brains of mzLrrk2 fish at the age of 11 mo. How loss of *lrrk2* underpins these findings is unclear. One possibility is that the increase of MAO-dependent monoamine catabolism be the consequence of impaired synaptic function in dopaminergic neurons (and, possibly, also serotonergic) as a result of problems at different levels of vesicle generation, trafficking, and fusion, all processes in which LRRK2 has been implicated [62]. Thus, if vesicles were “leaky”, or their dynamics impaired [28], this might lead to increased free dopamine, which is a substrate of MAO. In this scenario, increased MAO activity might be, at first, a protective mechanism against dopamine accumulation, as excess dopamine represents an oxidative threat due to toxic quinone formation [63]. However, while removing excess dopamine, MAO also generates toxic 3,4-dihydroxyphenylacetaldehyde and hydrogen peroxide, being potentially deleterious in the long term [64]. Therefore, MAO expression in dopaminergic (and serotonergic) neurons might cause selective vulnerability of these cell types in mzLrrk2 fish, particularly at late stages. In line with this contention, testing synaptic transmission in mzLrrk2 fish, possibly with and without MAO inhibitors [41], might help clarifying the functional association between Lrrk2 and MAO and provide a mechanistic insight. Lastly, to validate this association in mammal systems, especially human, will be crucial to assess safety of LRRK2 inhibitors as therapeutics against PD. If LRRK2 pharmacologic inhibition caused, in the long-term, increased degradation of monoamines such as dopamine and serotonin, then disease symptoms would be exacerbated, rather than attenuated.

To summarize, our results show that loss of *lrrk2* can compromise specific vertebrate brain functions, including MAO-dependent monoamine catabolism. Future efforts should aim at investigating whether loss of *lrrk2* selectively impairs synaptic transmission in monoaminergic neurons and how presence of Lrrk2 determines MAO expression and activity. To this aim, the generation of reporter lines in the *lrrk2*-null background might provide insightful tools. Thus, our zebrafish *lrrk2* knockout model offers a unique possibility to study the consequences of LRRK2 LOF *in vivo* and provides a means for identifying targets of interest in a fish-to-mammal translational perspective.

## Methods

### Ethics statement

All animal experiments were conducted according to the guidelines and under the supervision of the Landesdirektion Dresden (permits AZ: 24D-9168.11-1/2008-3; AZ: 24–9168.11-1/2013-5; AZ: TVV 24–9168.11-1/2013-14; TVV 46/2017; DD24-5131/346/11; DD24-5131/346/12). All efforts were made to minimize animal suffering and the number of animals used.

### Sequence alignment analyses

Sequences were retrieved from the latest available assemblies of the human and zebrafish genomes (GRCh38p.7 and GRCz10, respectively). Alignment analyses were performed using Clustal Omega [65] and BLAST [66].

### Cas9 and gRNA construction

*Cas9* mRNA and gRNAs were synthesized as previously described [67]. Briefly, *Cas9* mRNA was synthesized by *in vitro* transcription using T3 mMESSAGE mMACHINE kit (Ambion). gRNAs were generated and purified using the MEGAshortscript T7 and *mir*Vana miRNA isolation kits (Ambion), respectively. Sequences of the genomic target sites and oligonucleotides for making gRNAs are listed in Table 3.

**Table 3:** gRNA target sites for *lrrk2* locus deletion.

Target site	oligonucleotide for annealing	
**GGGATGTTAATGTGTCAACA** *TGG*	TA**GGGATGTTAATGTGTCAACA**	AAAC**TGTTGACACATTAACATC**
*CCG* **CCATCGACAACTCCCTGTCA**	TA**GGACAGGGAGTTGTCGATGG**	AAAC**CCATCGACAACTCCCTGT**

Sequences are oriented 5’ to 3’. Target site PAMs are italicized and underscored. Sequences present in the gRNAs are bold.

### MO oligonucleotides

MOs were handled according to the manufacturer’s instructions (Gene Tools). For the preparation of 1 mM stock solutions, MOs were diluted in distilled water, briefly heated to 65°C, and their concentration determined in 0.1 N HCl. Afterwards they were aliquoted and stored at –80°C. Prior to injection, MO stock solutions were thawed, briefly heated to 65°C, and then diluted with Danieau buffer and 10x Fast Green to appropriate working solutions, which were stored at 4°C. The MO oligonucleotides used were: TGACATTAAGGAACGCTAACCTTCC, targeting the *lrrk2* exon 10-intron 10 junction (E10I10); and TGTGTGTGCTTACGCTCCCTGGAAG, targeting the exon 12-intron 12 junction (E12I12).

### TILLING and TALEN screening

Targeting induced local lesion in genomes (TILLING) mutagenesis screening was performed as previously described [46]. Outer (F1, R1) and inner (F2, R2) primers used are reported in Table 4. During the screening the allele *hu3557*, a splice mutation affecting the splice donor of *lrrk2* exon 27 (c.3972+2T>C) was recovered and designated as “*tud112*”. We also generated an additional, early stop codon-carrying *lrrk2* allele (c.1980_1990del) using transcription activator-like effector nucleases (TALENs), denominated *tud115*, which however could give rise to a truncated Lrrk2 product from a downstream alternative translation initiation site, and was therefore not considered further.

**Table 4:** Primers used.

Primer sequence	target	application
F: GGATGAAGAGTGGAATGAGGAGGAC R: ACTAAACTTGCTCGCAGACCC	*lrrk2*	validation of E10I10 MO
F: CGTGAAGGTTTTCCAAGCTGCT R: GACACACTGGTTCTTCAACACCTG	*lrrk2*	validation of E12I12 MO
F: TACAAGTGGGCCCGACTGGAGAAAC R: ATCCAGAGGCAGATCCCACAGATGC	*lrrk2*	genotyping for the hu3557 allele
F1: TGCGAGCGCTGTCTGCTGTTAC R1: TGTCTTTGCTCCTGACGGGCCA F2: TACACAGGCGCCAACATGACCG R2: AGCTACACGCTGGACTTGGGGT	*lrrk2*	genotyping for the *tud113* allele
F1: TCTGCTGAACTGGATGTTGGC R1: TAATCAAACCCCACGGCACC	*lrrk1*	ISH probe 1
F2: GGAGCCCAGTGAAAAAAACCC R2: GAACGGTGATGCGAGACGAC	*lrrk1*	ISH probe 2
F3: TCCTGTGTTTGGCAGCTCAGAATG R1	*lrrk1*	ISH probe 3
F: CGTGAAGGTTTTCCAAGCTGCT R: CTAACTCCCACAATCCCCTTCTTC	*lrrk2*	ISH probe 1
F: GGTCTTTTGGCTGCTGGTTGTAAC R: CGGTTTGGGTTTGGTGTCAATG	*lrrk2*	ISH probe 2
F: CTTCAACATGGAGGACTGCG R: CGTGAGGGGAAGTCTGTCAT	*lrrk1*	RT-qPCR
F: GGACCAGTCTAGACCGATGG R: CAAAATGTGTCCCGCTCTCG	*lrrk2*	RT-qPCR
F1: **TGTTTCCCAGTATTGGGTTG** R1: **GCAGATGGCGCTCTAGG** F2: TGTAAAACGACGGCCAGT**GCTGGAATAGTTGGTGGTTC** R2: AGGAAACAGCTATGACCAT**TAACCTGTCCGCAATAACAC**	*lrrk2*	TILLING screening^a^
F: TATGCTCGTGTCCTGGGATC R: CAAGACCCTGCCAAACTGTG	*mao*	RT-qPCR
F: CCTTCCTGGGTATGGAATCT R: GACAGCACTGTGTTGGCATA	*actb1*	RT-qPCR
F: GTGCCCATCTACGAGGGTTA R: TCTCAGCTGTGGTGGTGAAG	*actb2*	RT-qPCR [68]

^a^Sequences present in the *lrrk2* gene are bold.

All primers target the indicated locus or flanking regions. Sequences are oriented 5’ to 3’. Primers used for genotyping were used on genomic DNA template; else, cDNA. Abbreviations: F, forward primer; R, reverse primer.

### Zebrafish husbandry and germ line mutagenesis

Zebrafish were raised and maintained as previously described [69]. Zebrafish embryos were obtained by natural spawnings of adult fish and staged according to hours post fertilization (hpf) or standard criteria [70]. The wild-type line used was AB. For CRISPR/Cas9-mediated mutagenesis, 150 ng/μL dual NLS-tagged zebrafish codon-optimized *cas9* mRNA/50 ng/μL gRNAs/0.2% phenol red were co-injected into fertilized eggs, the embryos raised to adulthood, crossed to AB wild-type fish and the resulting F1 embryos screened by PCR. The identified mutation (c.−61_*42del; see Results section) was designated as “*tud113*”.

### Genotyping

Genotyping was performed using genomic DNA from individual or pooled embryos/larvae or fin clips from larvae or adult fish. Primers used are listed in Table 4. For genotyping for the *lrrk2*^*tud113*^ allele, see Results section. For genotyping for the *lrrk2*^*tud112*^ allele, see S4 Fig.

### Animal experiments

Fin clipping for genotyping purposes was performed on adult fish following anesthesia with 0.02% tricaine. Drug treatments are summarized in Table 5.

**Table 5:** Summary of drug treatments.

Drug	dose and solvent	exposure
BrdU	for larvae: 5 mM, E3 medium	60 min
EdU	for adults: 5 mM, fish water	12 h

All drugs were purchased from Sigma. Abbreviations: BrdU, 5-bromo-2’-deoxyuridine; EdU, 5-ethynyl-2’-deoxyuridine.

### Behavioral analyses

The ZebraBox and ZebraCube apparatus were used in combination with the Viewpoint Application Manager software. All recordings were performed on individually isolated animals between 2–6 pm. Larvae were transferred into 24-well plates the day before recording. Each well was internally lined with Parafilm to minimize reflection and filled with 750 μL E3 medium, changed daily. Animals whose tracking was lost by the software for over 20% of the total recording time were excluded from the analyses. To quantify spontaneous swimming and thigmotaxis, adult fish were lodged in opaque cylindrical boxes (ø = 80 mm) filled with 100 mL fish facility water, else in opaque parallelepipedal boxes (l×w = 190×80 mm) filled with 500 mL fish facility water. Each recording was preceded by 10 min acclimatization inside the apparatus. Spontaneous swimming was assessed for 10 min (integration period: 600 s) in the dark under infrared light. Appropriate speed thresholds were chosen based on developmental stage: 2–10 mm/s for larvae; 2–40 mm/s for adults. Based on the speed thresholds, three swimming phases were defined: inactive phase, below the lower threshold; normal swimming phase, between the lower and upper threshold; bursting phase, above the upper threshold. For each swimming phase, three parameters were considered: entry count, duration (s), and distance swum (mm). Thigmotaxis was assessed using the same recordings of spontaneous swimming activity. To this aim, the recording arena was digitally subdivided into an outer and inner area (for larvae ø = 15.6/10.6 mm; for adults ø = 80/55 mm). Scototaxis was assessed for 10 min in half-black, half-white parallelepipedal boxes. Olfactory function was assessed by delivering a stimulus in either of the shorter sides of parallelepipedal boxes. The stimulus consisted in 0.6 mL of an amino acid mix (Ala, Cys, His, Lys, Met, Val, 0.1 mM each) delivered through a syringe pump (1.5 mL/min). Fish were starved for 24 h before the experiments. Fish behavior was recorded 5 min before and 5 min after stimulus delivery. For every 1 min of recording, a preference index was defined [71] as $\frac{ts-tc}{ts+tc}$, where *ts* is the time spent in the stimulus side, *tc* the time spent in the control side.

### Tissue processing and histochemistry

Tissue processing is summarized in Table 6. Bleaching was performed using 3% hydrogen peroxide/0.1% Tween20/1% potassium hydroxide. Clearing [72] was carried out overnight at room temperature. The procedure caused slight swelling of tissue, more pronounced in younger samples. IHC was performed as previously described on whole embryos/larvae [73], dissected larval brains [41], and cryosections [74]. Primary antibodies used are listed in Table 7. Secondary antibodies (1:500) were conjugated to Alexa Fluor 488, 555, 633, 700 (Invitrogen). For combined staining with one or more antibodies requiring antigen retrieval, the following precautions were used: HuC/D IHC was performed first if combined with BrdU IHC, otherwise last; TH(1) IHC was performed last. TUNEL assay on larval brains was carried out using the ApopTag Red or Fluorescein In Situ Apoptosis Detection Kits (Millipore) according to manufacturer’s instructions with the following adjustments: tissue was washed 3×10 min with sodium citrate/Triton X-100 0.1% in PBS prior to acetic acid/ethanol post-fixation; incubation in the equilibration buffer was carried out for 1 h at room temperature. When combined with IHC, TUNEL assay was performed first. EdU detection was performed using the Click- iT EdU Alexa Fluor 488 Imaging Kit (Fisher Scientific) according to manufacturer’s instructions. When combined with IHC, EdU detection was performed first. ISH and probe generation were performed on embryos and at least three adult individuals [75]. All synthesized and individually tested ISH probes (Table 4) showed a redundant pattern. The *mao* probe was provided by P. Panula’s laboratory. Monoamine oxidase histochemistry (MAO HC) was carried out as previously described [41] with the following adjustments: staining was developed for 90 min; stained tissue was post-fixed in 4% paraformaldehyde. For MAO HC on adult brain sections, fish were transcardially perfused with fixative.

**Table 6:** Summary of tissue processing.

Tissue	application	fixation	bleaching	clearing
embryos (24–72 hpf)	IHC, ISH	4% PFA	yes (from 32 hpf)	no
larvae (sections, 5 dpf)	IHC, ISH	4% PFA	no	no
larval brains (5 dpf)	IHC	2% PFA/1% DMSO	yes	yes
larval brains (5 dpf)	MAO HC	4% EDAC/0.4% NHS/1% DMSO	no	no
adult heads (sections)	IHC, ISH	4% PFA	no	no
adult heads (sections)	MAO HC	4% EDAC/0.4% NHS/1% DMSO	no	no

Abbreviations: DMSO, dimethyl sulfoxide; EDAC, 1-ethyl-3-(3-dimethylaminopropyl)carbodiimide; NHS, *N*-hydroxysuccinimide; PFA, paraformaldehyde.

**Table 7:** Primary antibodies used.

antigen, host (company)	antigen retrieval	dilution
acetylated Tubulin, mouse IgG_2b_ (Sigma)	no	1:1,000
BrdU, rat (Serotec)	2 N hydrochloric acid for 20 min at 37°C, followed by 5 min was in 0.1% sodium tetraborate buffer, pH = 8.5	1:500
Claudin k, rat [76]	no	1:1,000
HuC/D, mouse IgG_2b_ (Molecular Probes)	Tris-HCl buffer, pH = 8.0 for 5 min at 98°C	1:200
L-Plastin, rabbit [77]^a^	no	1:5,000
pH3, rabbit (Millipore)	no	1:200
TH(1), mouse IgG_1_ (Immunostar)	for larval brains: no for adult brains: 10 mM sodium citrate buffer, pH = 6.0 for 15 min at 85°C	1:1,000 1:1,000
TH(1/2), rabbit [59,78]	for larval brains: no for adult brains: no, unless in combination with BrdU: 10 mM sodium citrate buffer, pH = 6.0 for 15 min at 85°C	1:1,000

^a^Expression plasmid provided by M.J. Redd.

Abbreviations: TH(1), tyrosine hydroxylase (isoform 1); TH(1/2), tyrosine hydroxylase (isoforms 1 and 2).

### High performance liquid chromatography measurements

Tissue samples consisted each in 10-pooled whole larvae or single adult brains. Larvae were starved 24 h before tissue collection to minimize possible contamination from amines in the gastrointestinal tract. An equal number of male and female adult fish were sacrificed. Tissue homogenization and catabolite measurements via electrochemical detection coupled with high performance liquid chromatography (HPLC) were performed as previously described [41].

### Immunoblotting

Protein pellets obtained from the tissue samples used in HPLC were homogenized by sonication at room temperature in 5% SDS in PBS, pH 7.4, to perform the BCA assay. The solutions were then diluted with a loading buffer stock containing Tris-HCl, pH 6.8, β-mercaptoethanol, glycerol, and bromophenol blue (final concentration: 0.0625 M Tris-HCl, 5% β -mercaptoethanol, 10% glycerol, and 0.002% bromophenol blue). The samples were heated at 95°C for 5 min and loaded on a gel for SDS-PAGE (4% stacking gel, 9% separating gel). The loading volume was adjusted in order to load 20 μg total protein per well. PageRuler Prestained protein markers (Thermo Scientific) were used to control protein separation. The proteins were electrophoretically transferred to an Immobilon P PVDF membrane in an eBlot device (GenScript) according to manufacturer’s instructions and the membrane was processed for immunoblotting in an Odyssey CLx system (Li-Cor) according to manufacturer’s instructions. Mouse monoclonal anti-TH(1) antibody (Immunostar) was used as the primary antibody (1:2,000), and a goat anti-mouse IRDye800 antibody (Li-Cor) was used as the secondary antibody (1:20,000). The fluorescent bands were quantitated by the ImageStudio software supplied with the Odyssey CLx system, and the membranes were stained with ProAct membrane stain (M282–1L, Amresco Inc.) as the loading control.

### MAO activity enzymatic assay

Peroxidase-linked colorimetric assay of MAO activity was performed as previously described [79].

### RNA extraction and RT-qPCR

For pooled embryos or individual brains, total RNA was extracted using TRIzol (Invitrogen); for fish oocytes, an RNA purification kit was used (Norgen). cDNA was synthesized using Superscript III First Strand Synthesis System (Invitrogen). Reverse transcriptase-quantitative PCR (RT-qPCR) was performed using the LightCycler 480 SYBR Green I Master mix and the LightCycler 480 Instrument (Roche). Target gene expression was normalized by *actb1* or *actb2* expression.

### Image acquisition and processing for analysis

Confocal images were acquired with: Zeiss LSM 780 upright confocal microscope using C-Apochromat 10x/0.45 W and LD LCI Plan-Apochromat 25x/0.8 Imm Corr DIC M27 objectives for water immersion; Leica-SP5 confocal microscope using 20x/0.7, 40x/0.75, or 63x/1.2 Water objectives. To minimize channel crosstalk, images were acquired sequentially. Maximum intensity projections of stacks were created using Fiji. Brain sections of larvae and juveniles were imaged using the Zeiss Axioscan.Z1 with the following setup: Zeiss Colibri 7 LED light source (DAPI channel: 395 nm LED with 10% intensity, 15ms exposure time, emission filter: 430–470 nm; red channel: 576 nm LED with 100/int., 15ms exposure time, emission filter: 580–611 nm) equipped with an Orca Flash camera (16 bit). Bright-field images were acquired with an Olympus DP71 or DP80 color cameras connected to an MVX10 microscope. All devices were provided by the BIOTEC/CRTD Light Microscopy Facility. Images were processed using Fiji [80]. Image processing such as white balance and brightness/contrast adjustments was applied linearly across entire images and equally to both mutants and controls. Quantification of myelinated Claudin k+ bundles in larval brains was carried out manually through whole stacks. Quantification of cells was carried out manually through whole stacks (larval brains) or sections (adult brains). To quantify HuC/D, acetylated Tubulin, and Claudin k staining, segmentation algorithms were applied to mask stained areas (Otsu’s method) in unprocessed coronal sections. For HuC/D staining, the percent stained area over total brain section area was measured (average of 4 sections). For acetylated Tubulin and Claudin k staining, the integrated density of the stained areas was measured (average of up to 4 sections for acetylated Tubulin, average of up to 5 sections for Claudin k). To quantify microglia/leukocyte and analyze their morphology in larval brains, confocal stacks were background-subtracted using the sliding paraboloid method, despeckled, and thresholded (Li’s method). Obvious artifacts were manually removed from subsequent processing and analyses. The 3D ImageJ Suite plugin [81] was used to segment objects (minimum size threshold: 1,000; objects on borders excluded) and extract morphological data from 3D masks. The same 3D masks were subsequently skeletonized and subjected to 3D skeleton analysis using the AnalyzeSkeleton plugin on Fiji [82]. Loops were pruned using the shortest branch method. For each skeleton, the longest shortest path was also calculated. A ramification index was defined as $\frac{2b}{j+e}$, where *b* is the number of branches, *j* the number of junctions, *e* the number of end-points, as previously defined [82]. For quantification of TH+/BrdU+ cells, whole larvae were sectioned and sections spread into four series; per each individual, TH+/BrdU+ cells were quantified in the brain in one series and the total number subsequently multiplied by 4, thus yielding an estimate of the total number of cells/brain (n/brain).

### Statistical analyses

To minimize experimental bias, several measures were taken. For sample allocation, simple randomization was used for both larvae and adults; when enough adult males were available for both experimental and control groups, male-only groups were preferred. To prevent batch effects, the same experimental procedures and conditions were applied to experimental and control groups. To exclude subjective bias, all manual quantifications were carried out blindly. All conclusions from qualitative data were based on at least three replicates/group. The data analyses for this paper were performed using: the Real Statistics Resource Pack software (Release for Mac 3.1.2, copyright 2013–2016) developed by C. Zaiontz (www.real-statistics.com); R [83]; and GraphPad Prism version 9.0 for Mac OS X. To compare means, requirements of normal distribution and homoscedasticity were checked using Shapiro-Wilk’s test and the F-test, respectively. To determine the statistical significance of group differences, *P* values were calculated using: Student’s *t*-test, for normally distributed and homoscedastic data; Student’s *t*-test with Welch’s correction, for normally distributed and heteroscedastic data; Mann-Whitney’s *U*-test for non-normally distributed data. For each experiment, sample sizes are reported in the Figures. Plot features are described in the Figure legends. Within the Figures, significant comparisons are marked by asterisks: *, *P*<0.0500; **, *P*<0.0100; ***, *P*<0.0010. *P* values rounded to four decimal digits are reported in the main text. All numerical data underlying graphs are provided in the S1 Data file.

## Supporting information

S1 Fig Expression of *lrrk2* during zebrafish development. (A) Maternal *lrrk2* at sphere stage. (B-D) Low ubiquitous expression at tailbud (B), 15-somites (C), and 20-somites stage (D). (E-G) Increasing expression in the central nervous system and tail muscles at 24 (E, F) and 28 hpf (G). (F) Cross section of the tail at the position indicated in (E). (H-K) Decreasing expression of *lrrk2* in the rostral somites and tail tip at 32 hpf (H) and progressive restriction to the head area (I and J), pectoral fin buds (J, arrows) and sensory organs (K, arrows) at 48 hpf. Black arrowheads mark areas of persistent expression in the head (H and I), tail tip (H), and spinal cord (H); hollow arrowheads mark areas of weak (H) or no expression (I). (L-O) Gradual restriction of *lrrk2* expression during eye development at 32 (L), 48 dpf (M), and 6 mo (N and O). In the adult eye, *lrrk2* is mainly expressed in the inner nuclear layer (O, white asterisk) and the ganglion cell layer (O, black asterisk). Abbreviations: GCL, ganglion cell layer; INL, inner nuclear layer; IPL, inner plexiform layer; ONL, outer nuclear layer; OPL, outer plexiform layer; PR, photoreceptors.
(TIF)

S2 Fig Expression of *lrrk2* in the adult brain. (A-N) Expression of *lrrk2* is detected throughout the adult brain from the olfactory bulb (A) to the rhombencephalon (N), albeit weakly, as reflected by the long chromogenic signal development time (up to 72 h). The location of the brain cross-sections is illustrated. (A and B) In the olfactory bulb, *lrrk2* is mainly expressed in the ICL and ECL (A) and at the interface with the emerging telencephalon (B). (B) Telencephalic expression is found in the Vd, Vc, and Vv regions, along the ventricular zone, weakly in the D region (C), including the subregions Dc, Dm, Dd and Dl, but strongly in the Vs region (D). (E-M) In the diencephalon, *lrrk2* is detected in the PPa, Hav, VM, and VL regions (E), Had, A, SC, PPp and weakly in the PM regions (F) and furthermore in the TPp (G), PPv, CP (H), PTN, and PG regions (I) and most posterior in the SG region (J). Expression continues ventrally in the hypothalamus in the Hv (I), Hc, Hd, DIL (J-M) and CM regions (L). In the midbrain, *lrrk2* signal is present in the PGZ (G-L), TL (H and I), DTN, EW (I and J), NLV (J) and TS regions (I and J), absent in the superior RF region (K). Cerebellar expression is seen in the granular layers of the *valvula cerebelli* (J), in the Cce (J-L), EG (L) and Lca regions (M), but not in the CC region (M) and only very weakly in the RF region (K). (N) Weak expression in the hindbrain is detected in parts of the *medulla oblongata*, more precisely in the LX and LVII. Abbreviations: A, anterior thalamic nucleus; ATN, anterior tuberal nucleus; Cantd, *commissura anterior*, *pars dorsalis*; Cantv, *commissura anterior*, *pars ventralis*; CC, cerebellar crest; Cce, cerebellar corpus; CM, mammillary body; Cpost, central posterior thalamic nucleus; D, dorsal telencephalic area; Dc, central zone of D; Dd: dorsal zone of D; DIL, diffuse nucleus of the inferior lobe; Dl, lateral zone of D; Dm, medial zone of D; DP, dorsal posterior thalamic nucleus; DTN, dorsal tegmental nucleus; ECL, external cellular layer of the olfactory bulb; EG, granular eminence; EW, Edinger-Westphal nucleus; GL, glomerular layer of olfactory bulb; Had, dorsal habenular nucleus; Hav, ventral habenular nucleus; Hc, caudal zone of periventricular hypothalamus; Hd, dorsal zone of periventricular hypothalamus; Hv, ventral zone of periventricular hypothalamus; ICL, internal cellular layer of the olfactory bulb; IRF, inferior reticular formation; Lca, caudal lobe of the cerebellum; LLF, lateral longitudinal fascicle; LVII, facial lobe; LX, vagal lobe; MLF, medial longitudinal fascicle; NLV, nucleus *lateralis valvulae*; PG, preglomerular area; PGc, caudal preglomerular nucleus; PGZ, periventricular grey zone of the optic tectum; PM, magnocellular preoptic nucleus; Ppa, parvocellular preoptic nucleus, *pars anterior*; PPp, parvocellular preoptic nucleus, *pars posterior*; PPv, periventricular pretectal nucleus, *pars ventralis*; PTN, posterior tuberal nucleus/posterior tuberculum; RF, reticular formation; SC, suprachiasmatic nucleus; SG, subglomerular nucleus; TeO, optic tectum; TL, *torus longitudinalis*; Tla, *torus lateralis*; TPM, *tractus pretectomamillaris*; TPp, periventricular nucleus of posterior tuberculum; TS, *torus semicircularis*; TSc, central nucleus of TS; TSvl, ventrolateral nucleus of TS; Vam, medial division of *valvula cerebelli*; Vc, central nucleus of the ventral telencephalic area; Vd, dorsal nucleus of the ventral telencephalic area; VL, ventrolateral thalamic nucleus; VM, ventromedial thalamic nucleus; Vs, supracommissural nucleus of the ventral telencephalic area; Vv, ventral nucleus of the ventral telencephalic area; VIII, octaval nerve.
(TIF)

S3 Fig Knockdown of zebrafish *lrrk2* using splice-inhibiting morpholinos. (A) Splice-inhibiting morpholinos (MOs) were designed to block the splice donor site at the 3’ end of *lrrk2* exon 10 (MO4) and exon 12 (MO5), resulting in an excision of the targeted exon on mRNA level, determining a frameshift and premature stop codon. For comparison, the target sites of published zebrafish *lrrk2*-directed MOs are also shown (green: reference [35]; grey: reference [37]; magenta: reference [36]; black: present study): two are translation-inhibiting (diamond arrowheads), the others splice-inhibiting (triangular arrowheads). The predicted effects of splice-inhibiting MOs on the protein level are shown: the position of the first affected amino acid is indicated by a magenta vertical line; the extent of the frameshift until translation stop is depicted by a horizontal grey line. MO7 is predicted to cause an in-frame deletion. Abbreviations: aa, amino acids; kbp, kilobase pairs. (B) Working concentrations (mM) for the exon 10-intron 10–11 junction (E10I10; MO4 in A) and exon 12-intron 12–13 junction (E12I12; MO5 in A) were determined via RT-PCR in comparison with uninjected (ui) controls. Complete splice inhibition was achieved using 0.125 mM of E10I10 (C) and 1.5 mM of E10I12 (D). Abbreviation: bp, base pairs.
(TIF)

S4 Fig Generation and characterization of the zebrafish *lrrk2*^*tud112*^ allele. (A) *N*-ethyl-*N*-nitrosourea-mediated mutagenesis was used to generate a *lrrk2*-null zebrafish line. The identified allele (*lrrk2*^*tud112*^) consisted in a T>C substitution (c.3972+2T>C) disrupting the splice donor site of *lrrk2* exon 27, causing the retention of the ensuing intron and a premature stop codon (p.(Ile1252AlafsTer9)). (B) The *tud112* mutation also disrupts an RsaI restriction site, allowing identification of mutation carriers via restriction fragment length polymorphism (RFLP)-PCR. To this aim, PCR primers (F and R in A) were designed to amplify a 253-base pairs-long product comprising the RsaI site: upon RsaI-mediated digestion, only the amplicon of the wild-type allele is cleaved into two fragments (198 and 55 base pairs; lower band not shown), allowing identification of wild-type (*lrrk2*^*+*^), heterozygous (*lrrk2*^*+/tud112*^), and homozygous mutant (*lrrk2*^*tud112*^) individuals in comparison to non-digested product (nd). Abbreviations: aa, amino acids; bp, base pairs. (C) Nonsense-mediated *lrrk2* RNA decay in maternal-zygotic *tud112* mutants (mzLrrk2^tud112^) demonstrated via ISH on 24-hpf embryos. (D) Reduced expression of *th1* CA marker in mzLrrk2^tud112^ embryos. ISH for the CA marker *th1* gene reveals correct development of CA cell clusters in terms of position and size (scale bar), but the overall *th1* expression appears reduced in maternal-zygotic *tud112* mutants (mzLrrk2^tud112^).
(TIF)

S5 Fig Neurogenesis is overall normal in 1-mo youngsters. (A-C) To label proliferating neurons, a 5 mM 5-ethynyl-2’-deoxyuridine (EdU) pulse was delivered for 12 h to youngsters (30 dpf), followed by a 7-day chase, after which brains were inspected for HuC/D+/EdU+ cells. (B and C) Representative images showing HuC/D/EdU double labeling in a brain section. (B) Scale bar: 100 μm. (D) Quantification of HuC/D+/EdU+ cells revealed comparable levels of neurogenesis in both mzLrrk2 and control brains. Abbreviations: OB, olfactory bulb; Tel, telencephalon. Statistical analysis: two-tailed Student’s *t*-test.
(TIF)

S6 Fig Myelination in the ventromedial hindbrain is normal in 1-mo youngsters. Claudin k immunoreactivity of the commissural fibers in the ventromedial hindbrain was quantified in mzLrrk2 youngsters and wt controls (1 mo). Scale bar: 100 μm.
(TIF)

S7 Fig Microglia/leukocyte morphology is altered in mzLrrk2 larvae. (A-D) Segmentation strategy for microglia/leukocytes in larval brains (5 dpf) after L-Plastin IHC (Methods, *Image acquisition and processing for analysis* section). Segmented objects were colored with Glasbey’s lookup table to render them maximally distinguishable from one another. (E) Representative segmented images. Original representative images are displayed in Fig 2I. Scale bars: 100 μm. (F-H) Morphological analysis of segmented cells revealed that microglia/leukocytes were larger (F), more extended (G), and more ramified (H) in mzLrrk2 brains. Plots represent means ± s.e.m. Statistical analyses: two-tailed Student’s *t*-test.
(TIF)

S8 Fig The number of microglia/leukocytes in the adult brain is comparable between mzLrrk2 fish and wt controls. L-Plastin+ microglia/leukocytes were manually quantified in the whole telencephalon as representative region of the adult brain (6 mo). Scale bar: 100 μm.
(TIF)

S9 FigAnalysis of anxiety levels and odor response.(A-C) Analysis of thigmotaxis at 5 dpf (B) and 6 mo (C). (A) Sample 1-min tracking showing normal swimming (green) and bursting (red) activities. For analysis of thigmotaxis, the same recordings for spontaneous swimming activity were used, with the tracking arena divided into an inner and outer zone, and the time spent in the inner zone was quantified. (B and C) Violin plots summarizing data distributions. Statistical analyses: two-tailed Mann-Whitney’s *U* test. (D) Analysis of scototaxis at 6 mo. Violin plots summarize data distributions. Statistical analysis: two-tailed Mann-Whitney’s *U* test. (E) Analysis of the response to an odorant stimulus (amino acid mixture) at 6 mo. The preference index was defined as $\frac{ts-tc}{ts+tc}$, where *ts* is the time spent in the stimulus side, *tc* the time spent in the control side. The plot represents means ± s.e.m. Statistical analyses: two-tailed Mann-Whitney’s *U* test.(TIF)

S10 Fig Homology between the zebrafish Lrrk1 and Lrrk2 paralogous proteins and expression of zebrafish *lrrk1* gene during embryonic development. (A) Alignment of the whole sequence and the individual domains of zebrafish Lrrk1 (XP_021333791) and Lrrk2 (NP_001188385) proteins revealed a middle-to-low degree of conservation. The percentages of identity (same residues at the same positions in the alignment) and similarity (identical residues plus conservative substitutions) are indicated. Abbreviations: see Fig 1A. (B-J) Expression of *lrrk1* during zebrafish embryonic development. (B and C) Early expression in the anterior prechordal plate (B and C, black arrowheads), weak in the polster at the 5-somites stage (ss; B, full arrowhead), strong in the hatching gland at 15 ss (C, full arrowhead). (C-J) Expression in sensory organ anlagen (C-E, hollow arrowheads), weak at 15 ss (C), progressively strong through 20 ss (D), 24 hpf (E), 32 hpf (F-H), and 48 hpf (I and J). Transient expression in the midbrain-hindbrain boundary at 20 ss (D, arrow), telencephalon (E, arrow), ventral hindbrain (E, asterisk), and proctodeum (E, arrowhead) at 24 hpf.
(TIF)

S11 Fig Loss of *lrrk2* does not affect *lrrk1* expression throughout development. (A) Downregulation of *lrrk1* in the oocytes of transheterozygous *tud113/tud112* fish. Plot represents means ± s.e.m. Statistical analysis: two-tailed Student’s *t*-test. (B) Unaltered expression of *lrrk1* in 4-cell embryos derived from incross of *tud113/tud112* transheterozygotes. (C) Unaltered expression of *lrrk1* in mzLrrk2 larvae (5 dpf) as measured in the whole body by RT-qPCR. Plot represents means ± s.e.m. Statistical analysis: two-tailed Student’s *t*-test. (D and E) Brain *lrrk1* expression is below detection as assessed by ISH, despite long chromogenic signal development time (up to 72 h), in both mzLrrk2 and wt control larvae (5 dpf; D), youngsters (1 mo; E-top), and adult fish (6.5 mo; E-bottom). (E) The telencephalon is displayed as representative brain region.
(TIF)

S12 Fig CA neurogenesis is normal during larval development. (A-C) CA neurogenesis was evaluated at 5 dpf upon labeling proliferating cells at 3 dpf. To do so, 3-dpf embryos were soaked in 5 mM BrdU solution for 60 mins and then chased for CA neurons 2 days later using the pan-TH antibody (TH). (B and C) Representative images showing TH/BrdU double labeling in the larval brain (white arrowheads). Scale bar: 100 μm. (D and E) Quantification of TH+ neurons (D) and TH+/BrdU+ neurons (E). Statistical analyses: two-tailed Student’s *t*-test.
(TIF)

S13 Fig Analysis of monoamine catabolism in whole larvae. (A) Simplified scheme of the catabolism of dopamine and serotonin in zebrafish. Each arrow represents a distinct enzymatically-catalyzed step. Grey arrows indicate the reactions catalyzed by the combined action of monoamine oxidase (MAO)/aldehyde dehydrogenase. Abbreviations: 3-MT, 3-methoxytyramine; 5-HIAA, 5-hydroxyindoleacetic acid; 5-HT, serotonin; AD, adrenalin; DA, dopamine; DOPA, 3,4-dihydroxyphenylacetic acid; HVA, homovanillic acid; NA, noradrenalin. (B) Levels of biogenic amines and their catabolites at 5 dpf. Plot represents means ± s.e.m. Statistical analyses: (DA, NA, AD, DOPAC, HVA, 5-HT, 5-HIAA) two-tailed Student’s *t*-test; (3-MT) two-tailed Mann-Whitney’s *U*-test.
(TIF)

S14 Fig Potential regional increase of *mao* gene expression and MAO enzymatic activity in the adult zebrafish brain. (A) Expression of *mao* gene in whole brains at 11 mo was comparable between mzLrrk2 fish and wt controls as assessed by RT-qPCR. Plot represents means ± s.e.m. Statistical analysis: Student’s *t*-test. (B) ISH revealed potential upregulation of *mao* transcripts in the ventral diencephalon in mzLrrk2 fish (11 mo) relative to wt controls. (C) ISH revealed potential upregulation of *mao* transcripts in the ventral and dorsal (insets) telencephalon in mzLrrk2^tud112^ fish (10.5 mo) relative to wt controls. (D) MAO enzymatic activity in whole brains at 11 mo was comparable between mzLrrk2 fish and wt controls. Plot represents means ± s.e.m. Statistical analysis: Student’s *t*-test. (E) Histochemical detection of MAO enzymatic activity revealed potential slight upregulation in the ventral diencephalon (cross-sections 2 and 3).
(TIF)

S1 Data GraphPad Prism file containing all numerical data underlying the graphs herein presented. (PZFX)
