# Supplementary figures and images for: Deletion of lrrk2 causes early developmental abnormalities and age-dependent increase of monoamine catabolism in the zebrafish brain
Source: PLoS Genet. 2021 Sep 13;17(9):e1009794. doi: 10.1371/journal.pgen.1009794 (PMC8459977; doi:10.1371/journal.pgen.1009794)

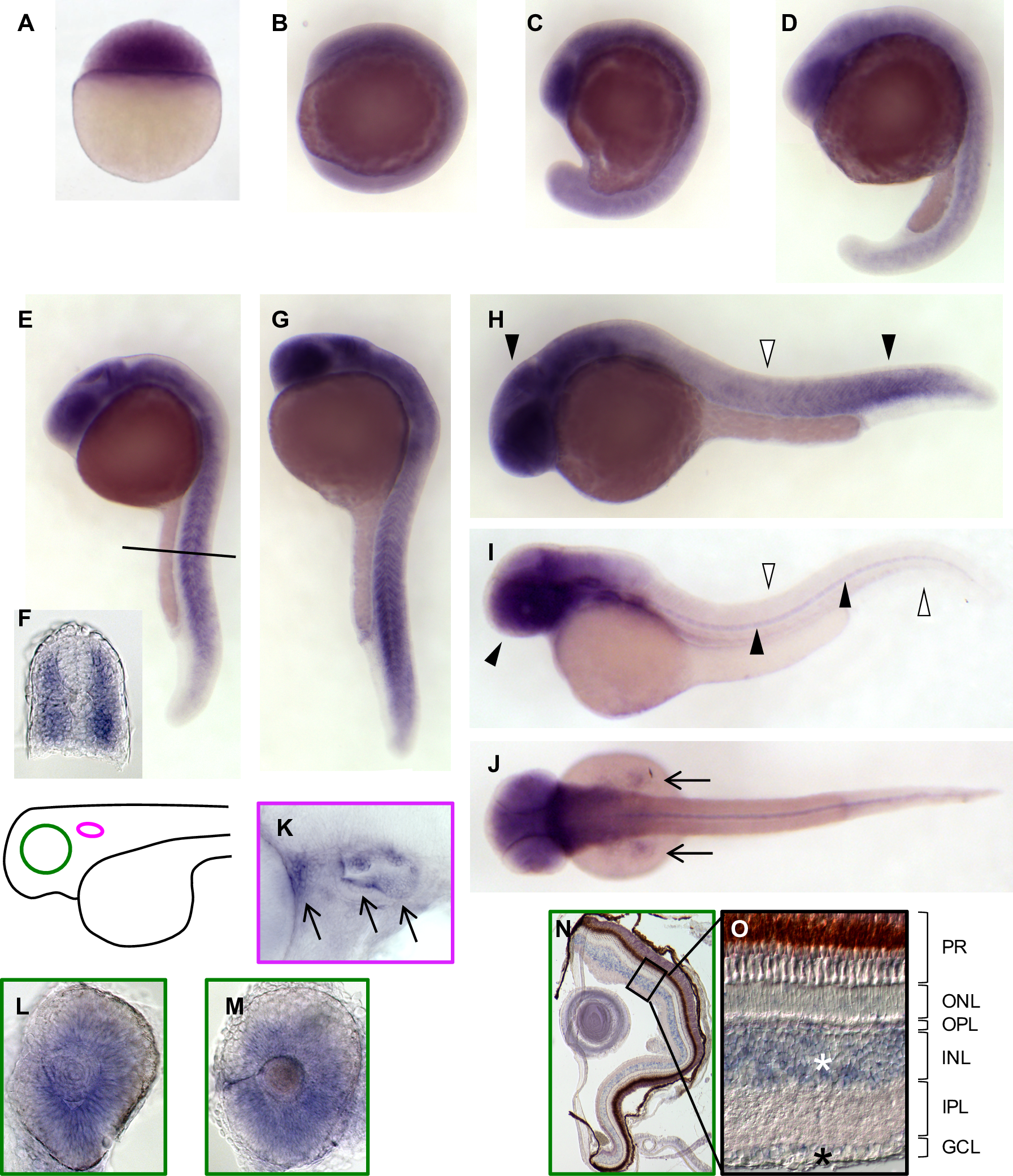

Supplement: S1 Fig — (A) Maternal lrrk2 at sphere stage. (B-D) Low ubiquitous expression at tailbud (B), 15-somites (C), and 20-somites stage (D). (E-G) Increasing expression in the central nervous system and tail muscles at 24 (E, F) and 28 hpf (G). (F) Cross section of the tail at the position indicated in (E). (H-K) Decreasing expression of lrrk2 in the rostral somites and tail tip at 32 hpf (H) and progressive restriction to the head area (I and J), pectoral fin buds (J, arrows) and sensory organs (K, arrows) at 48 hpf. Black arrowheads mark areas of persistent expression in the head (H and I), tail tip (H), and spinal cord (H); hollow arrowheads mark areas of weak (H) or no expression (I). (L-O) Gradual restriction of lrrk2 expression during eye development at 32 (L), 48 dpf (M), and 6 mo (N and O). In the adult eye, lrrk2 is mainly expressed in the inner nuclear layer (O, white asterisk) and the ganglion cell layer (O, black asterisk). Abbreviations: GCL, ganglion cell layer; INL, inner nuclear layer; IPL, inner plexiform layer; ONL, outer nuclear layer; OPL, outer plexiform layer; PR, photoreceptors. (TIF) [file pgen.1009794.s001.tif]

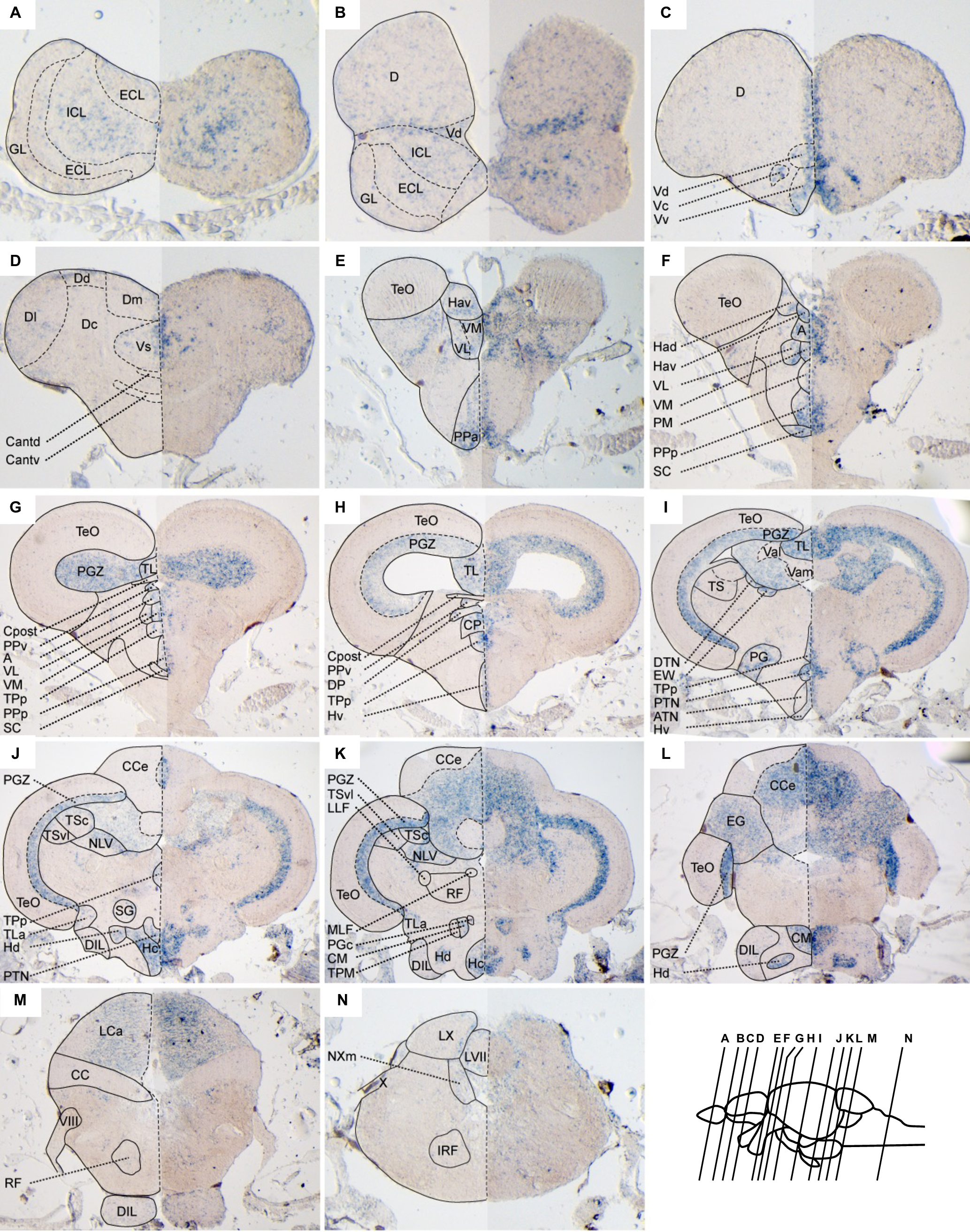

Supplement: S2 Fig — (A-N) Expression of lrrk2 is detected throughout the adult brain from the olfactory bulb (A) to the rhombencephalon (N), albeit weakly, as reflected by the long chromogenic signal development time (up to 72 h). The location of the brain cross-sections is illustrated. (A and B) In the olfactory bulb, lrrk2 is mainly expressed in the ICL and ECL (A) and at the interface with the emerging telencephalon (B). (B) Telencephalic expression is found in the Vd, Vc, and Vv regions, along the ventricular zone, weakly in the D region (C), including the subregions Dc, Dm, Dd and Dl, but strongly in the Vs region (D). (E-M) In the diencephalon, lrrk2 is detected in the PPa, Hav, VM, and VL regions (E), Had, A, SC, PPp and weakly in the PM regions (F) and furthermore in the TPp (G), PPv, CP (H), PTN, and PG regions (I) and most posterior in the SG region (J). Expression continues ventrally in the hypothalamus in the Hv (I), Hc, Hd, DIL (J-M) and CM regions (L). In the midbrain, lrrk2 signal is present in the PGZ (G-L), TL (H and I), DTN, EW (I and J), NLV (J) and TS regions (I and J), absent in the superior RF region (K). Cerebellar expression is seen in the granular layers of the valvula cerebelli (J), in the Cce (J-L), EG (L) and Lca regions (M), but not in the CC region (M) and only very weakly in the RF region (K). (N) Weak expression in the hindbrain is detected in parts of the medulla oblongata, more precisely in the LX and LVII. Abbreviations: A, anterior thalamic nucleus; ATN, anterior tuberal nucleus; Cantd, commissura anterior, pars dorsalis; Cantv, commissura anterior, pars ventralis; CC, cerebellar crest; Cce, cerebellar corpus; CM, mammillary body; Cpost, central posterior thalamic nucleus; D, dorsal telencephalic area; Dc, central zone of D; Dd: dorsal zone of D; DIL, diffuse nucleus of the inferior lobe; Dl, lateral zone of D; Dm, medial zone of D; DP, dorsal posterior thalamic nucleus; DTN, dorsal tegmental nucleus; ECL, external cellular layer of the olfactory bul [file pgen.1009794.s002.tif]

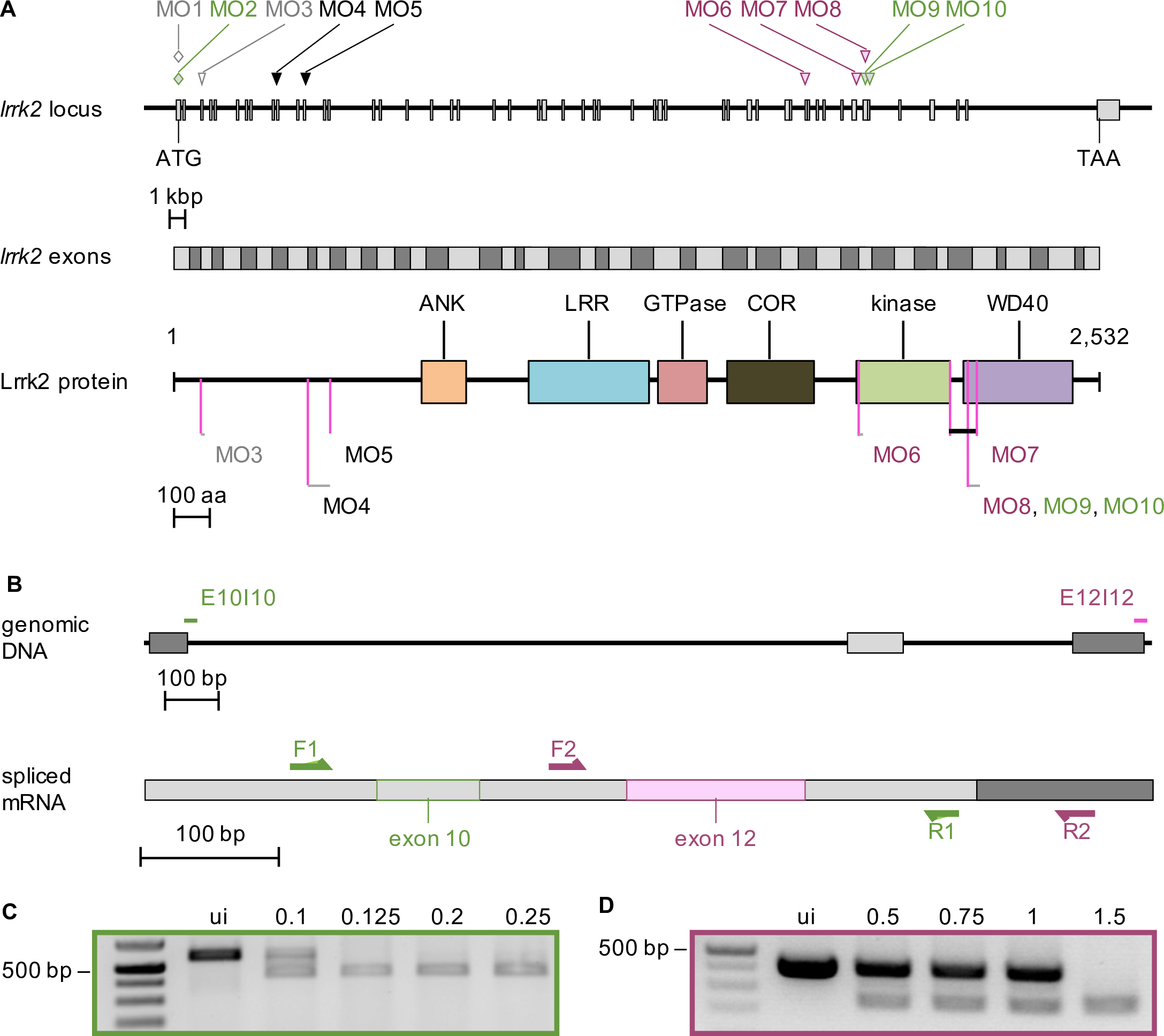

Supplement: S3 Fig — (A) Splice-inhibiting morpholinos (MOs) were designed to block the splice donor site at the 3’ end of lrrk2 exon 10 (MO4) and exon 12 (MO5), resulting in an excision of the targeted exon on mRNA level, determining a frameshift and premature stop codon. For comparison, the target sites of published zebrafish lrrk2-directed MOs are also shown (green: reference [35]; grey: reference [37]; magenta: reference [36]; black: present study): two are translation-inhibiting (diamond arrowheads), the others splice-inhibiting (triangular arrowheads). The predicted effects of splice-inhibiting MOs on the protein level are shown: the position of the first affected amino acid is indicated by a magenta vertical line; the extent of the frameshift until translation stop is depicted by a horizontal grey line. MO7 is predicted to cause an in-frame deletion. Abbreviations: aa, amino acids; kbp, kilobase pairs. (B) Working concentrations (mM) for the exon 10-intron 10–11 junction (E10I10; MO4 in A) and exon 12-intron 12–13 junction (E12I12; MO5 in A) were determined via RT-PCR in comparison with uninjected (ui) controls. Complete splice inhibition was achieved using 0.125 mM of E10I10 (C) and 1.5 mM of E10I12 (D). Abbreviation: bp, base pairs. (TIF) [file pgen.1009794.s003.tif]

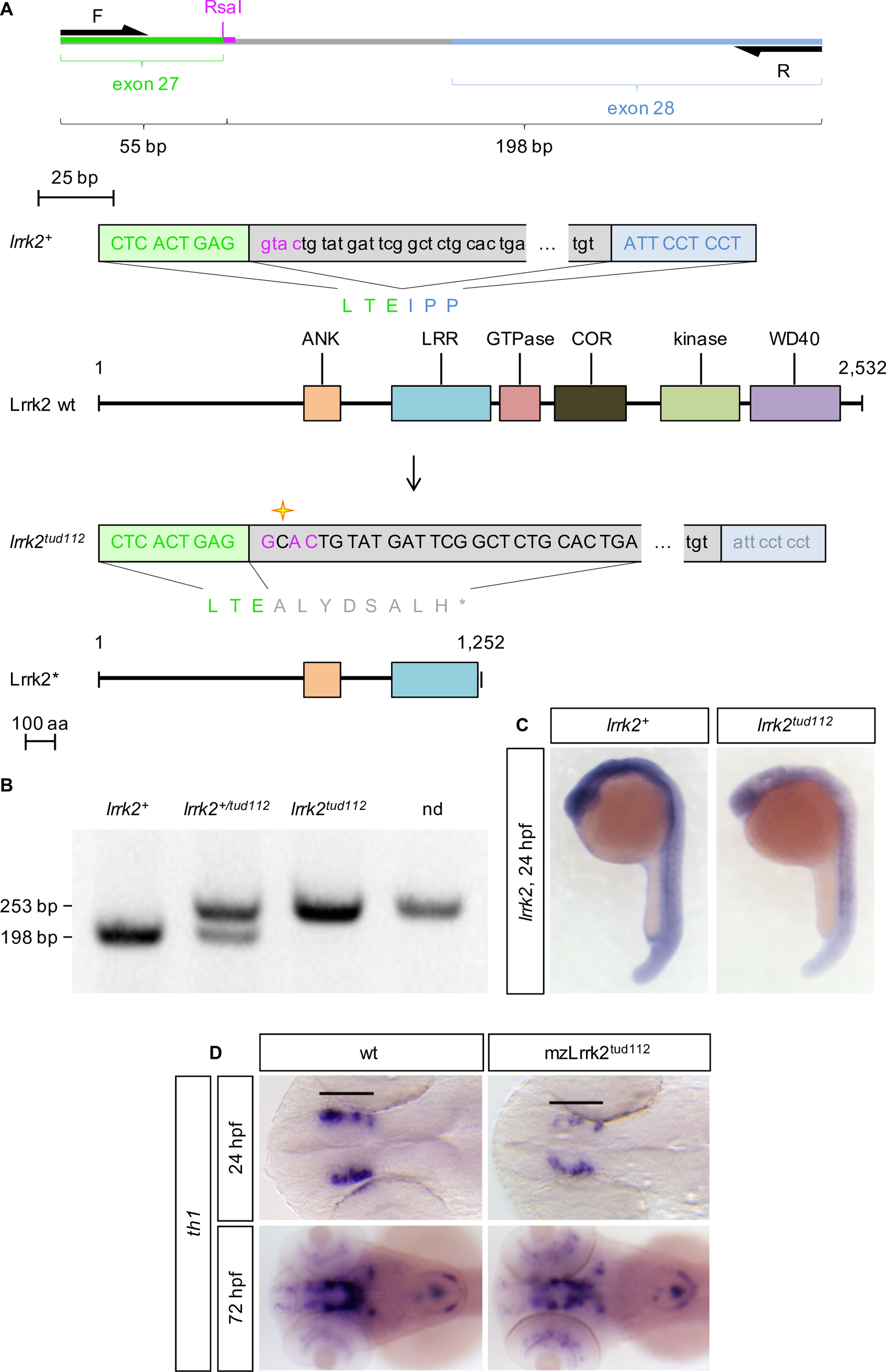

Supplement: S4 Fig — (A) N-ethyl-N-nitrosourea-mediated mutagenesis was used to generate a lrrk2-null zebrafish line. The identified allele (lrrk2tud112) consisted in a T>C substitution (c.3972+2T>C) disrupting the splice donor site of lrrk2 exon 27, causing the retention of the ensuing intron and a premature stop codon (p.(Ile1252AlafsTer9)). (B) The tud112 mutation also disrupts an RsaI restriction site, allowing identification of mutation carriers via restriction fragment length polymorphism (RFLP)-PCR. To this aim, PCR primers (F and R in A) were designed to amplify a 253-base pairs-long product comprising the RsaI site: upon RsaI-mediated digestion, only the amplicon of the wild-type allele is cleaved into two fragments (198 and 55 base pairs; lower band not shown), allowing identification of wild-type (lrrk2+), heterozygous (lrrk2+/tud112), and homozygous mutant (lrrk2tud112) individuals in comparison to non-digested product (nd). Abbreviations: aa, amino acids; bp, base pairs. (C) Nonsense-mediated lrrk2 RNA decay in maternal-zygotic tud112 mutants (mzLrrk2tud112) demonstrated via ISH on 24-hpf embryos. (D) Reduced expression of th1 CA marker in mzLrrk2tud112 embryos. ISH for the CA marker th1 gene reveals correct development of CA cell clusters in terms of position and size (scale bar), but the overall th1 expression appears reduced in maternal-zygotic tud112 mutants (mzLrrk2tud112). (TIF) [file pgen.1009794.s004.tif]

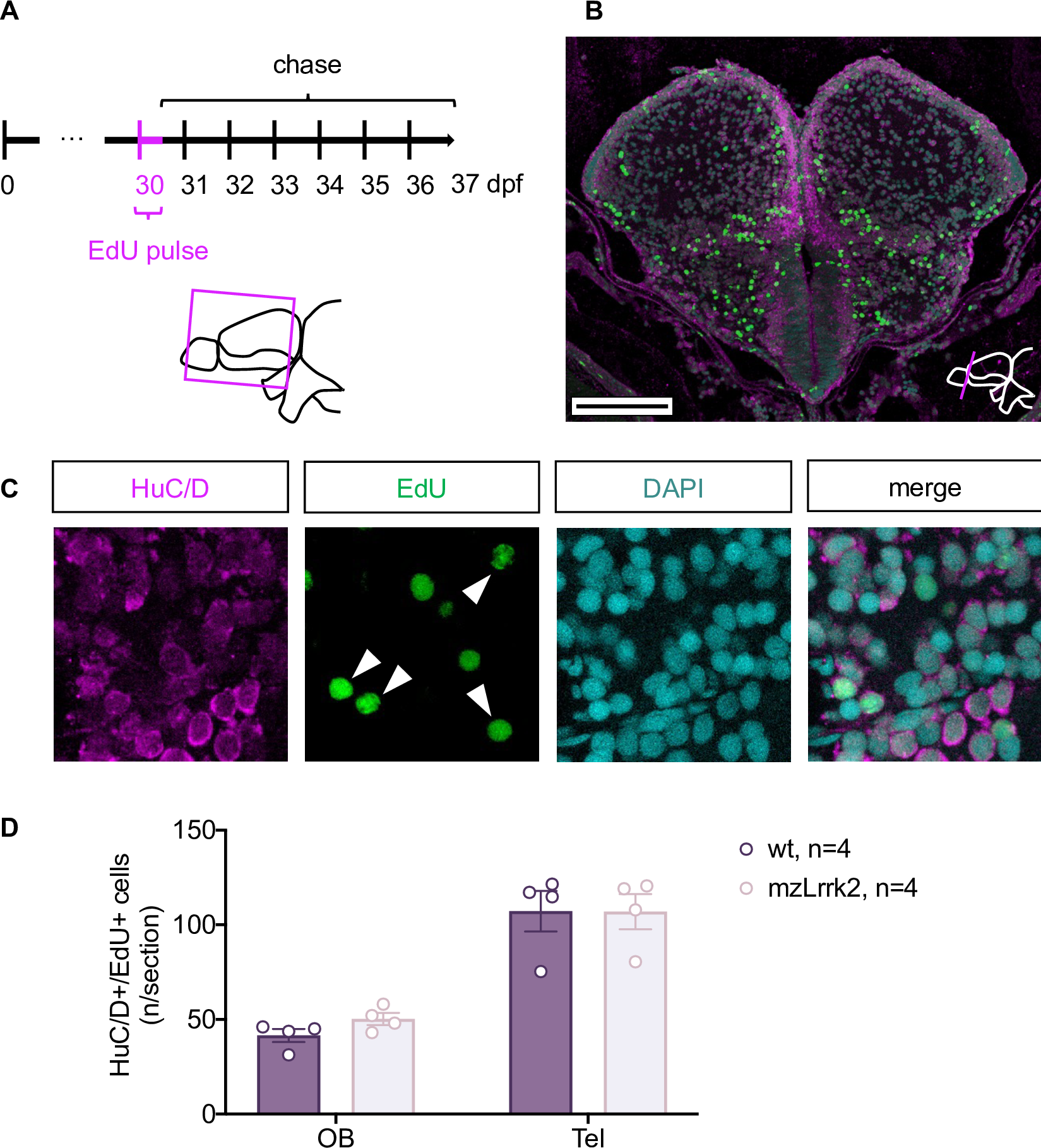

Supplement: S5 Fig — (A-C) To label proliferating neurons, a 5 mM 5-ethynyl-2’-deoxyuridine (EdU) pulse was delivered for 12 h to youngsters (30 dpf), followed by a 7-day chase, after which brains were inspected for HuC/D+/EdU+ cells. (B and C) Representative images showing HuC/D/EdU double labeling in a brain section. (B) Scale bar: 100 μm. (D) Quantification of HuC/D+/EdU+ cells revealed comparable levels of neurogenesis in both mzLrrk2 and control brains. Abbreviations: OB, olfactory bulb; Tel, telencephalon. Statistical analysis: two-tailed Student’s t-test. (TIF) [file pgen.1009794.s005.tif]

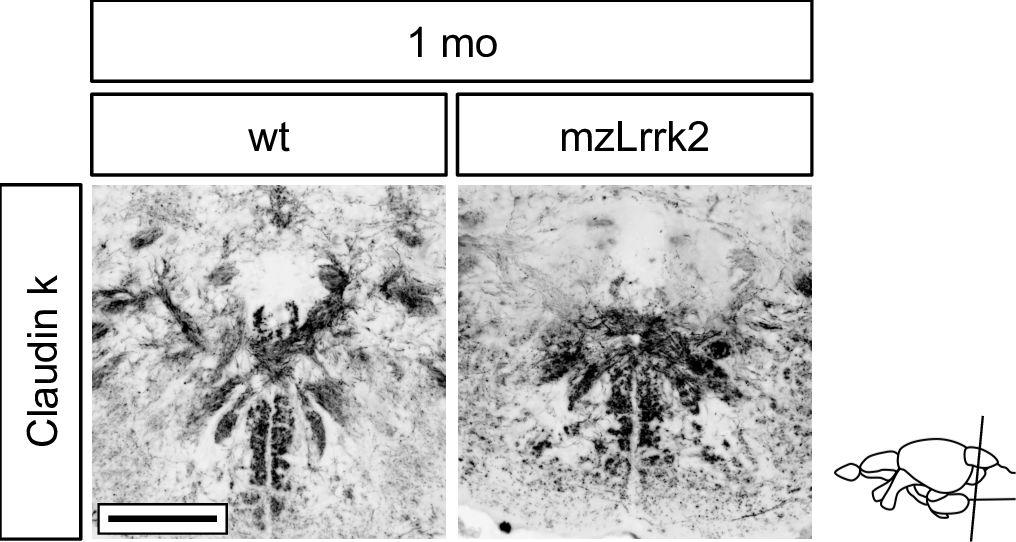

Supplement: S6 Fig — Claudin k immunoreactivity of the commissural fibers in the ventromedial hindbrain was quantified in mzLrrk2 youngsters and wt controls (1 mo). Scale bar: 100 μm. (TIF) [file pgen.1009794.s006.tif]

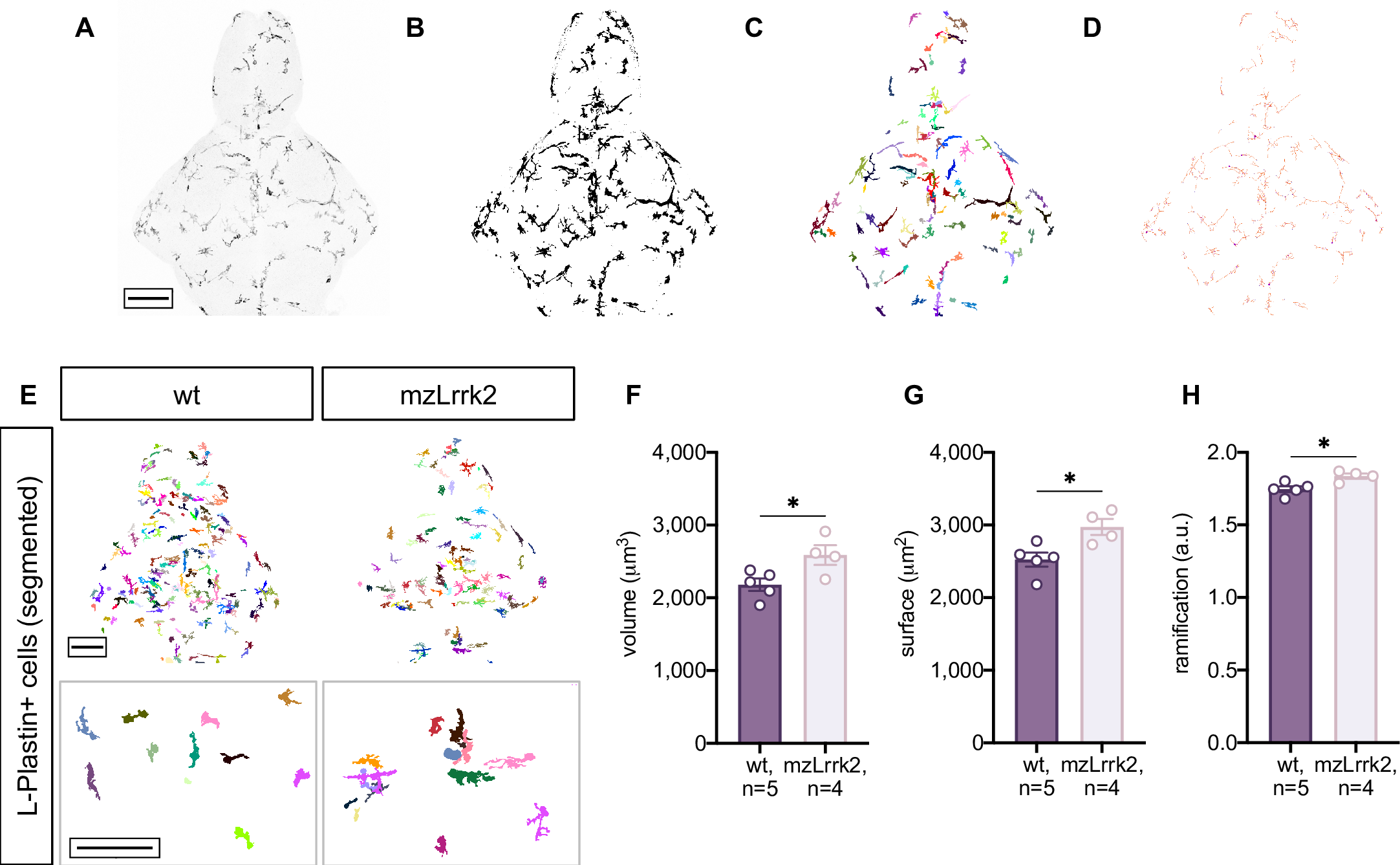

Supplement: S7 Fig — (A-D) Segmentation strategy for microglia/leukocytes in larval brains (5 dpf) after L-Plastin IHC (Methods, Image acquisition and processing for analysis section). Segmented objects were colored with Glasbey’s lookup table to render them maximally distinguishable from one another. (E) Representative segmented images. Original representative images are displayed in Fig 2I. Scale bars: 100 μm. (F-H) Morphological analysis of segmented cells revealed that microglia/leukocytes were larger (F), more extended (G), and more ramified (H) in mzLrrk2 brains. Plots represent means ± s.e.m. Statistical analyses: two-tailed Student’s t-test. (TIF) [file pgen.1009794.s007.tif]

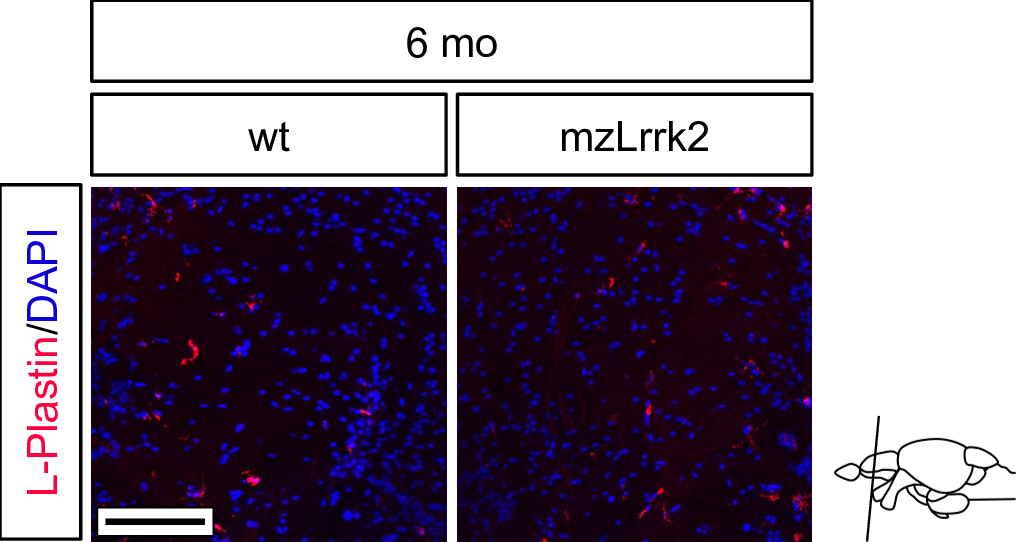

Supplement: S8 Fig — L-Plastin+ microglia/leukocytes were manually quantified in the whole telencephalon as representative region of the adult brain (6 mo). Scale bar: 100 μm. (TIF) [file pgen.1009794.s008.tif]

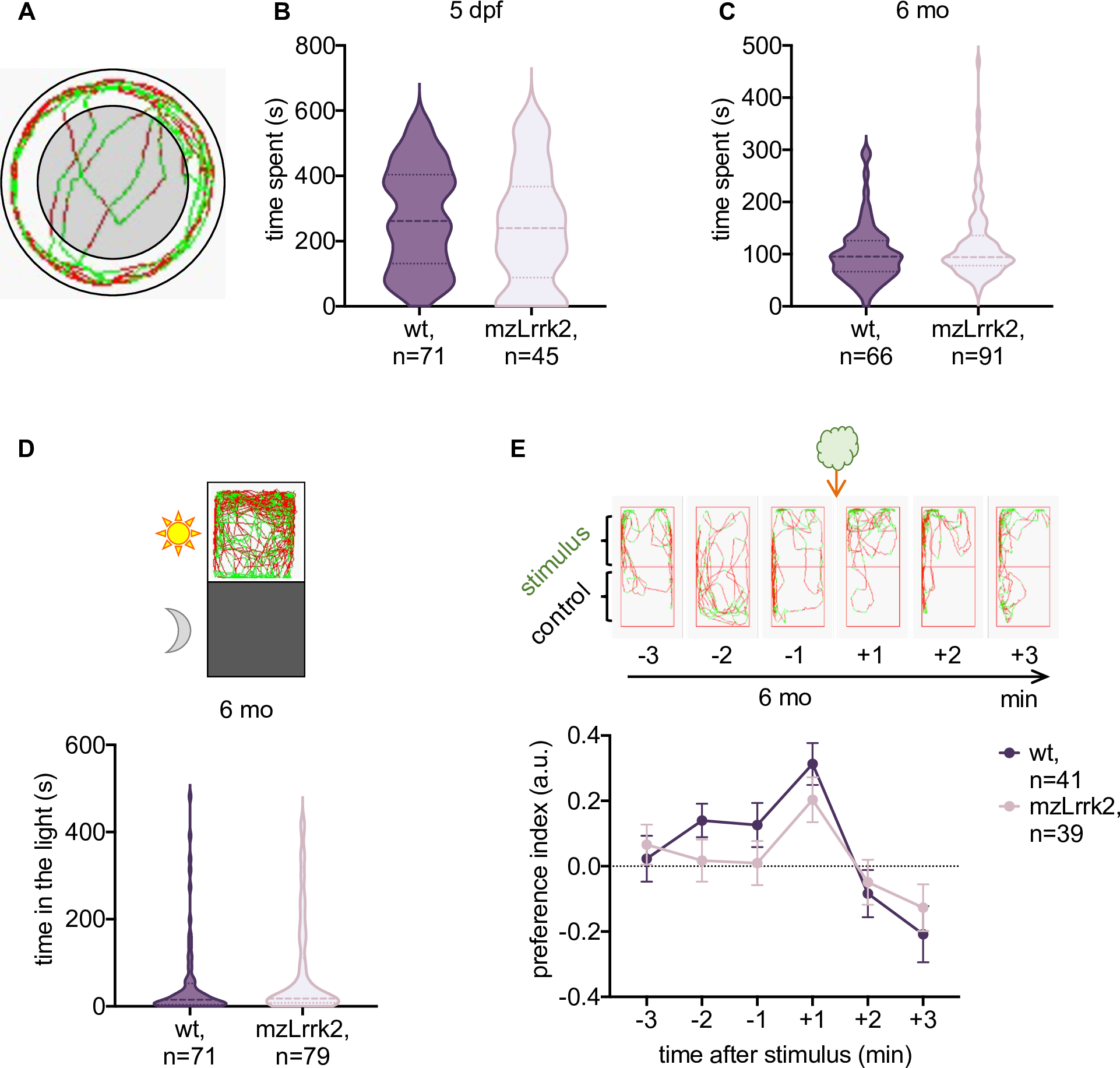

Supplement: S9 Fig — (A-C) Analysis of thigmotaxis at 5 dpf (B) and 6 mo (C). (A) Sample 1-min tracking showing normal swimming (green) and bursting (red) activities. For analysis of thigmotaxis, the same recordings for spontaneous swimming activity were used, with the tracking arena divided into an inner and outer zone, and the time spent in the inner zone was quantified. (B and C) Violin plots summarizing data distributions. Statistical analyses: two-tailed Mann-Whitney’s U test. (D) Analysis of scototaxis at 6 mo. Violin plots summarize data distributions. Statistical analysis: two-tailed Mann-Whitney’s U test. (E) Analysis of the response to an odorant stimulus (amino acid mixture) at 6 mo. The preference index was defined as ts−tcts+tc, where ts is the time spent in the stimulus side, tc the time spent in the control side. The plot represents means ± s.e.m. Statistical analyses: two-tailed Mann-Whitney’s U test. (TIF) [file pgen.1009794.s009.tif]

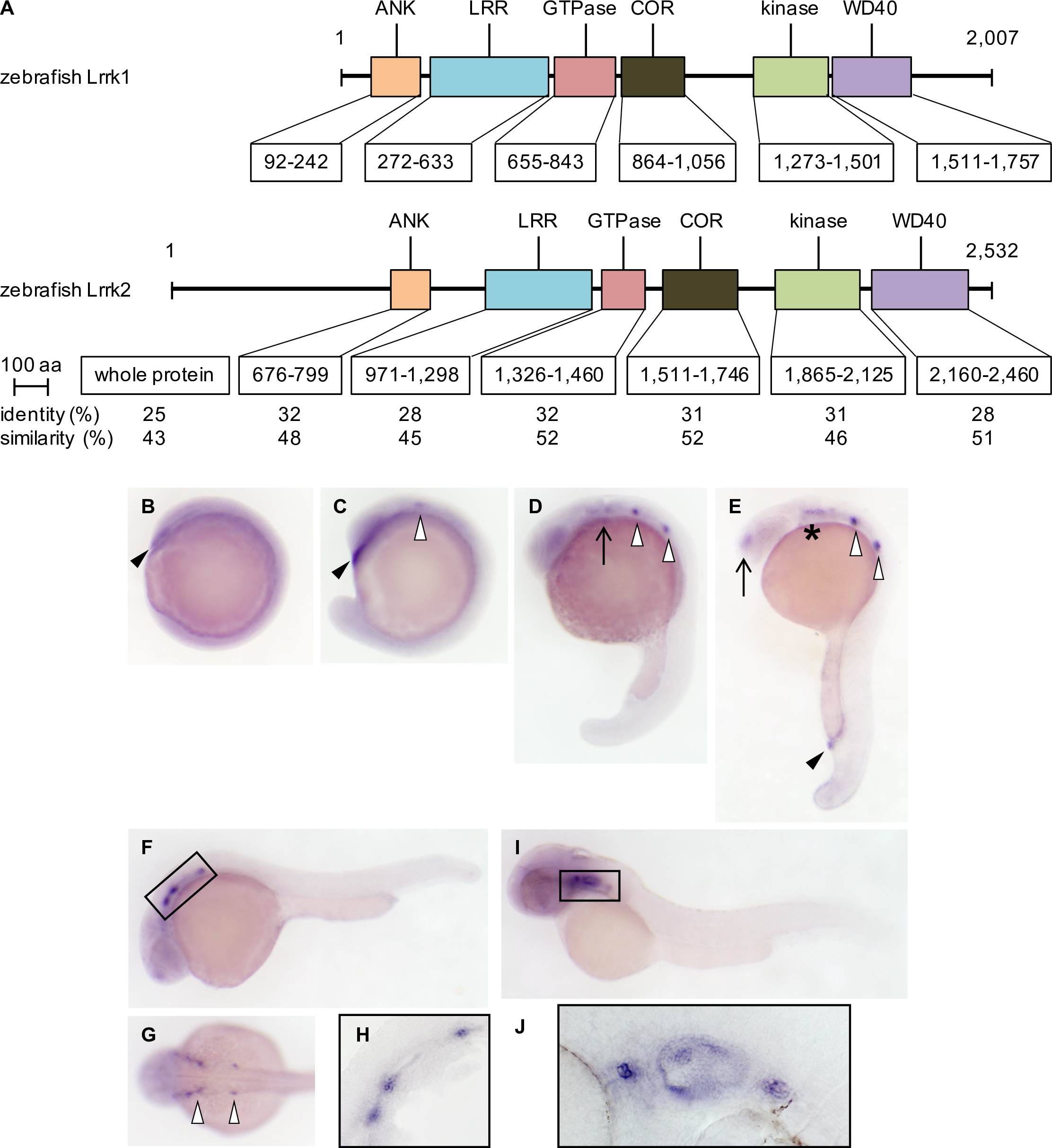

Supplement: S10 Fig — (A) Alignment of the whole sequence and the individual domains of zebrafish Lrrk1 (XP_021333791) and Lrrk2 (NP_001188385) proteins revealed a middle-to-low degree of conservation. The percentages of identity (same residues at the same positions in the alignment) and similarity (identical residues plus conservative substitutions) are indicated. Abbreviations: see Fig 1A. (B-J) Expression of lrrk1 during zebrafish embryonic development. (B and C) Early expression in the anterior prechordal plate (B and C, black arrowheads), weak in the polster at the 5-somites stage (ss; B, full arrowhead), strong in the hatching gland at 15 ss (C, full arrowhead). (C-J) Expression in sensory organ anlagen (C-E, hollow arrowheads), weak at 15 ss (C), progressively strong through 20 ss (D), 24 hpf (E), 32 hpf (F-H), and 48 hpf (I and J). Transient expression in the midbrain-hindbrain boundary at 20 ss (D, arrow), telencephalon (E, arrow), ventral hindbrain (E, asterisk), and proctodeum (E, arrowhead) at 24 hpf. (TIF) [file pgen.1009794.s010.tif]

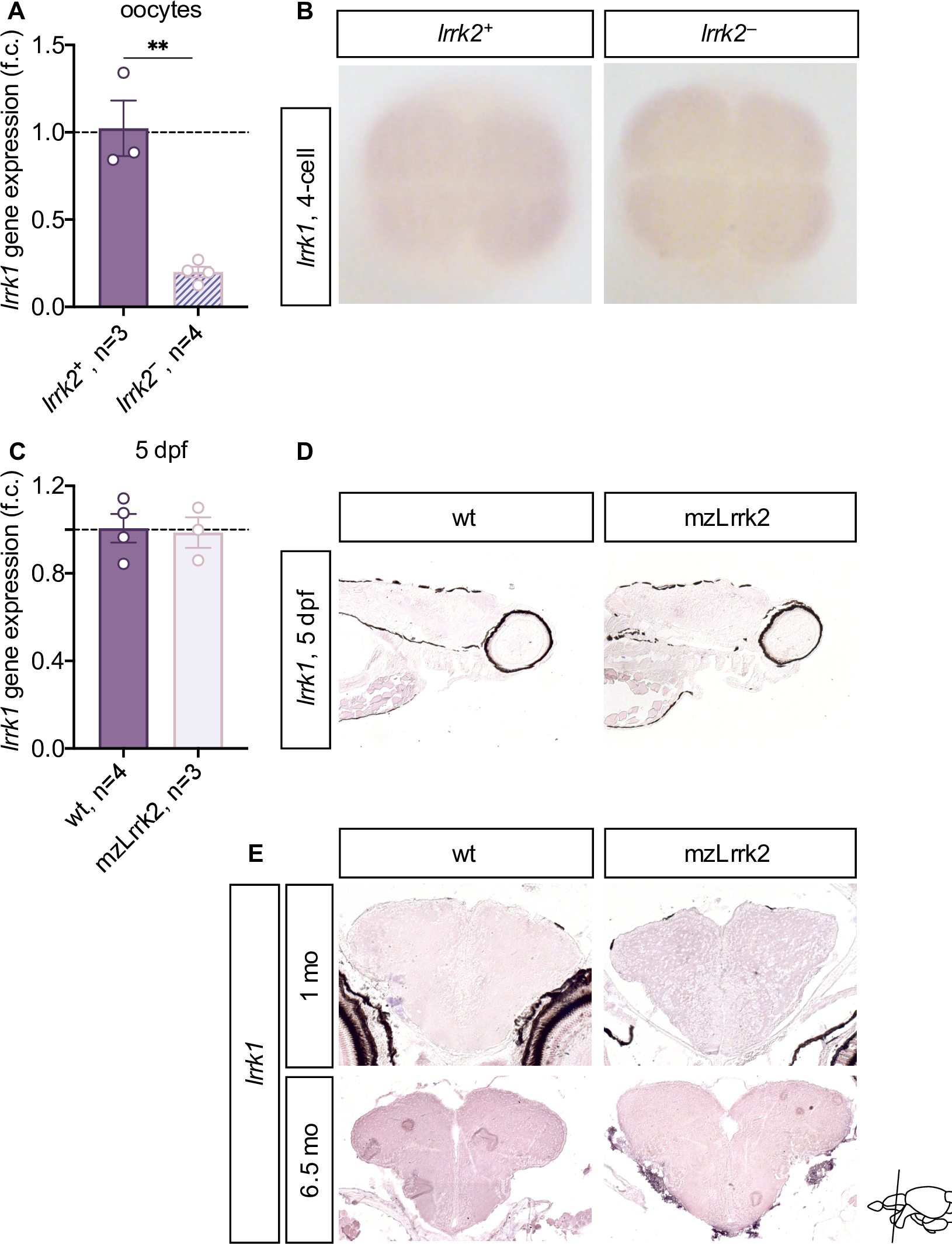

Supplement: S11 Fig — (A) Downregulation of lrrk1 in the oocytes of transheterozygous tud113/tud112 fish. Plot represents means ± s.e.m. Statistical analysis: two-tailed Student’s t-test. (B) Unaltered expression of lrrk1 in 4-cell embryos derived from incross of tud113/tud112 transheterozygotes. (C) Unaltered expression of lrrk1 in mzLrrk2 larvae (5 dpf) as measured in the whole body by RT-qPCR. Plot represents means ± s.e.m. Statistical analysis: two-tailed Student’s t-test. (D and E) Brain lrrk1 expression is below detection as assessed by ISH, despite long chromogenic signal development time (up to 72 h), in both mzLrrk2 and wt control larvae (5 dpf; D), youngsters (1 mo; E-top), and adult fish (6.5 mo; E-bottom). (E) The telencephalon is displayed as representative brain region. (TIF) [file pgen.1009794.s011.tif]

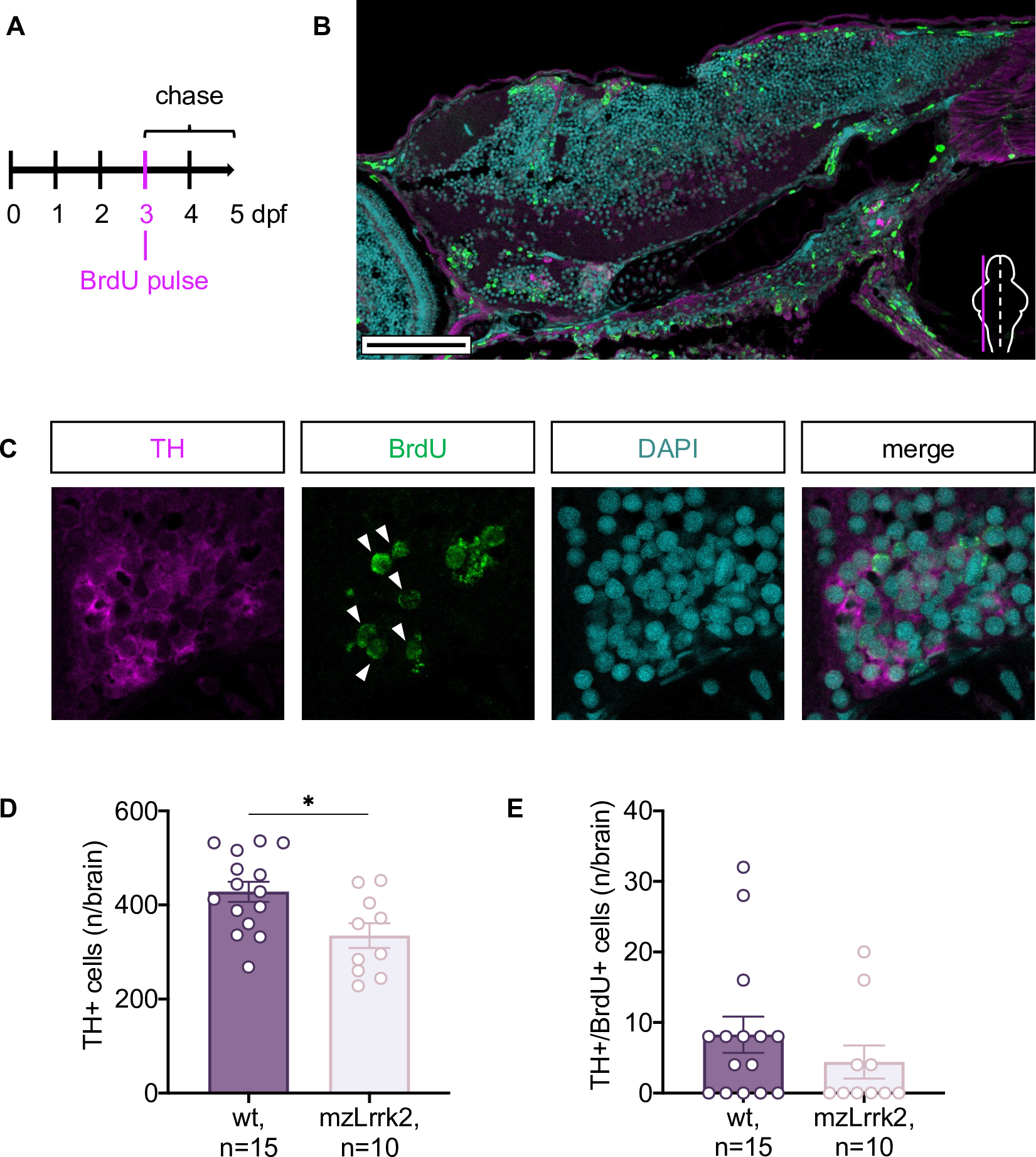

Supplement: S12 Fig — (A-C) CA neurogenesis was evaluated at 5 dpf upon labeling proliferating cells at 3 dpf. To do so, 3-dpf embryos were soaked in 5 mM BrdU solution for 60 mins and then chased for CA neurons 2 days later using the pan-TH antibody (TH). (B and C) Representative images showing TH/BrdU double labeling in the larval brain (white arrowheads). Scale bar: 100 μm. (D and E) Quantification of TH+ neurons (D) and TH+/BrdU+ neurons (E). Statistical analyses: two-tailed Student’s t-test. (TIF) [file pgen.1009794.s012.tif]

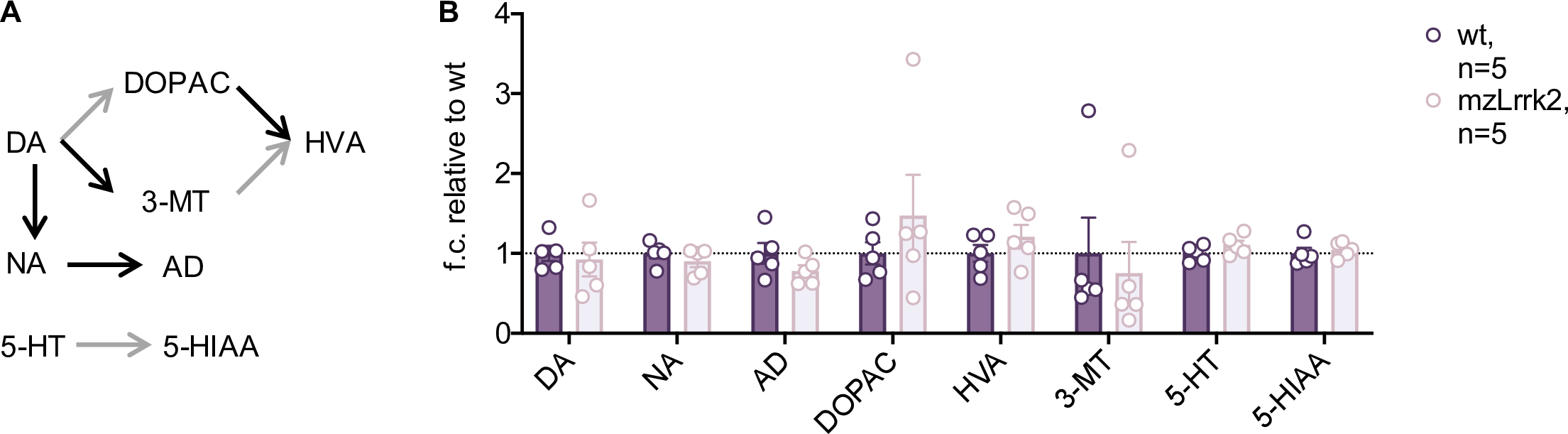

Supplement: S13 Fig — (A) Simplified scheme of the catabolism of dopamine and serotonin in zebrafish. Each arrow represents a distinct enzymatically-catalyzed step. Grey arrows indicate the reactions catalyzed by the combined action of monoamine oxidase (MAO)/aldehyde dehydrogenase. Abbreviations: 3-MT, 3-methoxytyramine; 5-HIAA, 5-hydroxyindoleacetic acid; 5-HT, serotonin; AD, adrenalin; DA, dopamine; DOPA, 3,4-dihydroxyphenylacetic acid; HVA, homovanillic acid; NA, noradrenalin. (B) Levels of biogenic amines and their catabolites at 5 dpf. Plot represents means ± s.e.m. Statistical analyses: (DA, NA, AD, DOPAC, HVA, 5-HT, 5-HIAA) two-tailed Student’s t-test; (3-MT) two-tailed Mann-Whitney’s U-test. (TIF) [file pgen.1009794.s013.tif]

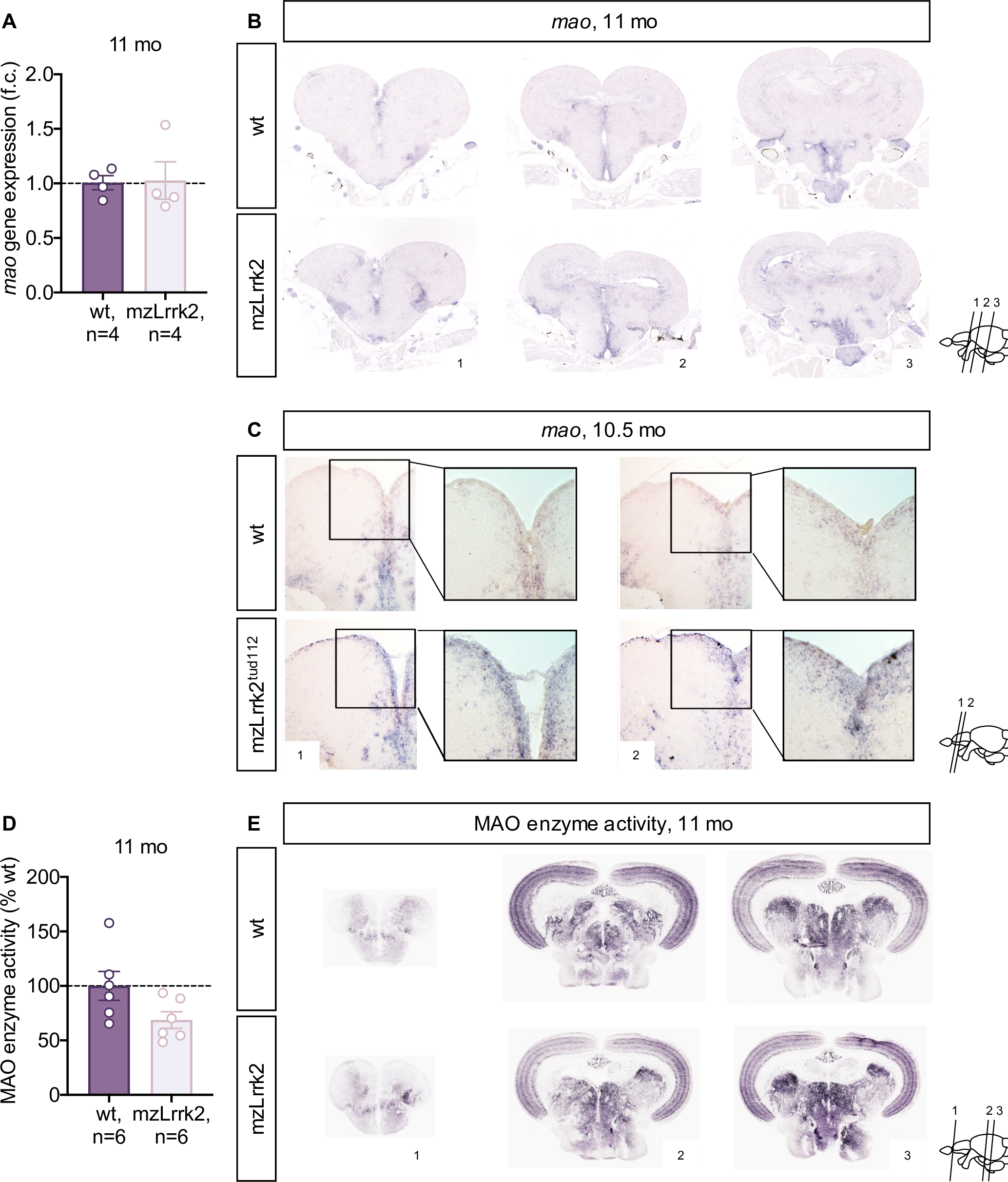

Supplement: S14 Fig — (A) Expression of mao gene in whole brains at 11 mo was comparable between mzLrrk2 fish and wt controls as assessed by RT-qPCR. Plot represents means ± s.e.m. Statistical analysis: Student’s t-test. (B) ISH revealed potential upregulation of mao transcripts in the ventral diencephalon in mzLrrk2 fish (11 mo) relative to wt controls. (C) ISH revealed potential upregulation of mao transcripts in the ventral and dorsal (insets) telencephalon in mzLrrk2tud112 fish (10.5 mo) relative to wt controls. (D) MAO enzymatic activity in whole brains at 11 mo was comparable between mzLrrk2 fish and wt controls. Plot represents means ± s.e.m. Statistical analysis: Student’s t-test. (E) Histochemical detection of MAO enzymatic activity revealed potential slight upregulation in the ventral diencephalon (cross-sections 2 and 3). (TIF) [file pgen.1009794.s014.tif]
